# Nursing health technologies in the prevention and control of hepatitis A: a scoping review

**DOI:** 10.1590/1980-220X-REEUSP-2025-0051en

**Published:** 2025-05-12

**Authors:** Leonardo Cassimiro Fonseca e Souza, Camila Stela Gomes, Laís Oliveira de Moraes Tavares, João Pedro Vasconcelos Paolinelli, João Marcos Alves Melo, Valéria Conceição de Oliveira, Brener Santos Silva, Gabriela Gonçalves Amaral

**Affiliations:** 1 Universidade do Estado de Minas Gerais, Divinópolis, MG, Brazil.; 2 Universidade Federal de São João del-Rei, Divinópolis, MG, Brazil.; 3 Universidade do Estado de Minas Gerais, Passos, MG, Brazil.; 4 Universidade de São Paulo, São Paulo, SP, Brazil.

**Keywords:** Hepatitis, Viral, Human, Hepatitis A, Disease Prevention, Communicable Disease Control, Nursing

## Abstract

**Objective:**

To map scientific evidence on nursing health technologies in the prevention and control of Hepatitis A.

**Method:**

Scoping review conducted in 2024 according to JBI recommendations and reported following PRISMA-ScR criteria. The search was carried out (10/06/2024) in databases and gray literature. The technologies found were classified as: soft, soft-hard, and hard. The results were analyzed descriptively and synthesized.

**Results:**

Ten studies were selected, all from the USA. Technologies include vaccination; serological testing; health education; reminders in health information systems; peer coaching training; case management; consultations; notification of diseases and injuries; clinical screening; and monitoring and tracking. Hard technologies prevailed.

**Conclusion:**

Primary Health Care stood out as the main focus, mainly addressing vaccination and health education. Secondary and Tertiary Care, in turn, focused on clinical screening and continuity of care. The concentration of studies of American origin reinforces the perception of literary insipience and possible negligence and lack of technical preparation in the prevention and control of Hepatitis A.

## INTRODUCTION

Hepatitis A is caused by the A virus, which belongs to the family *Picornaviridae* and the gender *Hepatovirus*, and is often called “infectious hepatitis”^(1)^. Symptoms, when present, are usually nonspecific, beginning with fatigue, malaise, fever, and muscle aches, followed by gastrointestinal manifestations such as nausea, vomiting, abdominal pain, constipation, or diarrhea. Dark urine may appear before jaundice. Symptoms usually appear 15 to 50 days after infection and tend to last less than two months^(2)^.

During the acute phase, initial viral replication takes place, followed by elimination of the virus in the feces. Transmissibility can occur from two weeks before to at least one week after the onset of jaundice or other clinical symptoms, in addition to elevated liver enzymes. The virus can be detected in feces about one to two weeks after exposure, persisting for an average of 79 days after the peak of infection^(3,4,5)^.

Infection in individuals over 50 years of age tends to progress in a more severe and symptomatic way, with jaundice in more than 70% of patients. The incubation period is, on average, 28 days, ranging from 15 to 50 days. Although there are no reports of hepatitis A leading to chronic hepatitis or hepatocellular carcinoma, cases of fulminant hepatitis may occur^(4,5)^. Complications are rare, the most serious being fulminant heart failure, which occurs in 0.5% of cases, in addition to extrahepatic manifestations, such as hemolysis, acalculous cholestasis, pleural and pericardial effusions, reactive arthritis, pancreatitis, and neurological manifestations^(1)^.

Hepatitis A is transmitted mainly through the oral-fecal route, associated with the consumption of contaminated food or water, and is influenced by low levels of basic sanitation and personal hygiene. Other forms of transmission include close personal contact (such as between household members or children in daycare centers), as well as sexual contact, especially through the oro-anal or digit-anal routes^(4)^. Among the populations at greatest risk of sexually transmitted infection, men who have sex with men (MSM) are a group that contributes to the increased incidence, including gay, bisexual, non-binary, and heterosexual men who have sexual relationships with other men^(6).^

Worldwide, there is a significant increase in the occurrence of sexually transmitted acute hepatitis A, especially among MSM. In the European Union, between 2016 and 2018, 19,947 cases were documented, which represents a fourfold increase compared to the previous three-year period (2012 to 2015)^(7)^. In Brazil, from 2000 to 2021, 718,651 confirmed cases of viral hepatitis were reported, of which 168,175 (23.4%) were hepatitis A. Despite the overall reduction in notifications, which reached 95.6% between 2011 and 2021^(8)^, this drop is largely due to the broad vaccination coverage provided free of charge to children up to 4 years, 11 months and 29 days^(9)^. However, in specific regions, such as the state of Rio de Janeiro, a significant increase was observed in 2018, with 9 cases per 100,000 inhabitants, in contrast to 0.3 cases per 100,000 inhabitants in the previous year. This increase was especially notable among the MSM population^(10)^.

Additionally, the transmission nature of hepatitis A is influenced by natural disasters that affect the distribution of food and water. The recurrence of floods, such as those that occurred in the state of Rio Grande do Sul in May 2024, favors the spread of the disease, highlighting sociodemographic factors that contribute to its propagation^(11)^. This underscores the urgency of implementing effective control and prevention measures.

Nursing care is fundamental in all infection control and prevention actions, covering several areas. This includes the viral hepatitis surveillance system, which monitors disease trends; the planning of prevention and control strategies; the performance of clinical screening of potential blood and blood product donors in blood banks; the administration of vaccines and immunization campaigns; the provision of assistance to pregnant women to prevent vertical transmission; the tracking of cases of viral hepatitis; the provision of tertiary care to individuals with acute and chronic forms of infections; and the production of knowledge that enables the safe implementation of evidence-based practices^(12)^.

It is important to note that hepatitis A is less addressed compared to other infections, such as hepatitis B, syphilis and HIV, among other sexually transmitted infections (STIs). This results in a significant lack of technical-practical knowledge and in the systematization of care for patients affected by this infection. Furthermore, there is a lack of protagonism and participation of nursing in the approach to hepatitis A, especially considering its sexual transmission and the specificities of morbidity and mortality. Therefore, nursing professionals should be involved in the prevention and promotion of health related to hepatitis A.

Thus, this study has as objetive to map scientific evidence on nursing health technologies in the prevention and control of Hepatitis A.

## METHOD

### Design of Study

This is a scoping review conducted in accordance with the recommendations of the *Joanna Briggs Institute* (JBI) – *Manual for Evidence Synthesis for Scoping review* ^(13)^; and reported following the criteria of *Preferred Reporting Items for Systematic reviews and Meta-Analyses extension for Scoping Reviews* (PRISMA-ScR)^(14)^. The review protocol is registered in the *Open Science Framework* (OSF) *Registries* (https://doi.org/10.17605/OSF.IO/MJESK).

### Data Sources and Research Strategy

A preliminary search was carried out in May 2024 in the OSF and PROSPERO databases of *National Institute for Health and Care Research*, to map and check similar studies in progress or completed, evaluating the scope and methodology used, without finding relevant records.

To define the guiding question, the mnemonic Population-Concept-Context (PCC) was used. The population (P) considered was nursing professionals; the concept (C), the technologies used in the prevention and control of hepatitis A; and the context (C), all levels of health care. Technologies were considered to be the different technical-practical skills used by nursing professionals during care, whether related to medical issues, education, epidemiological surveillance, prevention and health promotion, or even physical and material technologies. Thus, the guiding question formulated was: “What are the health technologies used by nursing professionals in the prevention and control of hepatitis A?”

Searches were carried out on the Virtual Health Library Portal (VHL), including the following databases: National Library of Medicine (NLM – MEDLINE)/National Library of Medicine and National Institutes of Health (PubMed); Latin American and Caribbean Health Sciences (LILACS); Índice *Bibliográfico Español en Ciencias de la Salud* (IBECS); and *Base de dados de Enfermagem* (BDEnf). Moreover, the following databases were checked: PubMed/Medline; EMBASE; Scopus; Web of Science (WOS); CINAHL Database; and Cochrane Library. For gray literature, the Catalog of Theses and Dissertations of the Coordination for the Improvement of Higher Education Personnel (CAPES) was consulted; Theses Canada; and WorldCat Dissertations and Theses. Finally, the references of the analyzed studies were checked to search for complementary literature.

The search was divided into stages: a) preliminary search to verify similar studies in progress, together with the identification of search terms and keywords; b) the gathering of terms and keywords, followed by the formulation of search strategies and their adaptation to the specificities of the databases consulted; c) verification of the results and selection of the records used. The search strategies, adjusted for each database, are published as supplementary material, available at https://doi.org/10.17605/OSF.IO/MJESK.

The inclusion of original articles with quantitative, qualitative or mixed approaches was defined; available in full; without time restrictions; in Portuguese, English, and Spanish; that addressed technologies used by nursing professionals in the prevention and control of hepatitis A. Experience reports were also included. Literature reviews, study protocols, letters, editorials, and posters were excluded. Furthermore, studies of a purely epidemiological nature, studies not related to the human population and/or studies dealing with food products were discarded.

### Data Collection and Extraction

After consulting the databases, carried out on 06/10/2024, the records obtained were imported into the software Rayyan, which assists in selection, starting with the removal of duplicates. Two independent reviewers performed screening by title and abstract. Based on this selection, the texts were analyzed in full to check for their relevance and compliance with the eligibility criteria, by two independent reviewers, following the double-blind review method. In case of impasses or doubts, a third reviewer was consulted. Of the records excluded due to full unavailability, an attempt was made to contact the authors of the studies, via *e-mail*, to request the work; however, there was no response. The PRISMA-ScR flow diagram^(14)^ was used to report search results and record the reasons for excluded articles.

A data collection instrument developed by the authors was used, which was evaluated through a pilot test with the extraction of data from five articles for calibration. The variables collected included: title, author(s), country, year of publication, objective(s), study design, participants, study setting, level of health care, technologies for hepatitis A prevention and control, technology classification, as well as conclusions and recommendations. Consistent with JBI recommendations^(15)^, individual analysis of the risk of bias of the selected studies was not performed.

To classify the technologies, the theoretical framework described by Merhy was followed^(16)^, being divided into soft technologies, which indicate those related to human relations, such as the production of bonds and relationships, autonomy, embracement, management of work processes; soft-hard, as in the case of well-structured knowledge, which operate in health work, such as internal medicine, psychoanalysis, epidemiology, among others; and hard, as in the case of technological equipment, machines, standards, organizational structures.

### Summary of Results

From the extracted data, a summary of the evidence was prepared, organized in tables, together with the interpretation and general synthesis of the findings, highlighting knowledge gaps. The contributions of available technologies were also raised, in addition to suggestions for public policies and the evolution of clinical nursing practice.

### Ethical Aspects

As it was a scoping review, the research was not submitted for consideration to a Research Ethics Committee.

## RESULTS

**Figure 1:**
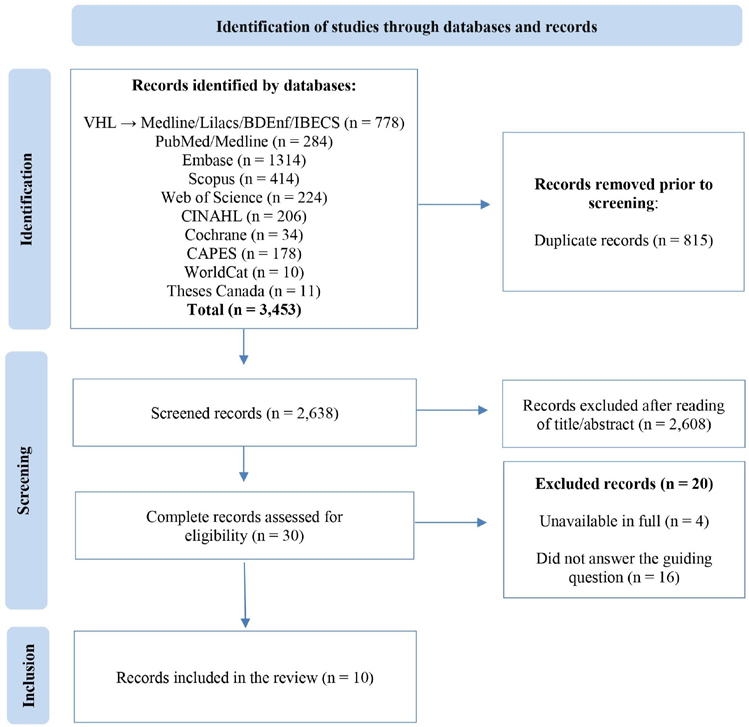
Flowchart of identification, selection, eligibility and inclusion of studies, based on PRISMA recommendations Divinópolis, MG, Brazil, 2024.

From searches in the databases, 3,453 records were retrieved. After excluding 815 duplicates, 2,638 files remained potentially eligible for review. Titles and abstracts were read to verify the eligibility criteria, resulting in the exclusion of 2,608 publications and 30 studies remaining for full reading. Finally, 10 studies were selected. The details of the search, identification, and selection stage of studies are described in Figure 1.

The studies were published between 2004 and 2022, with the highest occurrence in 2015 (n = 2; 20%) and 2020 (n = 2; 20%). Regarding nationality, in total, publications occurred in the United States, with emphasis on the states of Pennsylvania (n = 3; 30%) and California (n = 2; 20%). Regarding the design of the studies, four (40%) were experience reports; two (20%) were cross-sectional studies; one (10%) was a quasi-experimental study; one (10%) was a randomized clinical trial; one (10%) was a qualitative descriptive study; and one (10%) was a retrospective cohort study. Regarding the levels of health care, Primary Health Care (PHC) (n = 5; 50%) and Secondary Health Care (n = 2; 20%) stood out. Furthermore, the intersection between PHC and Secondary Health Care (n = 1; 10%) and PHC and Tertiary Healthcare (n = 1; 10%) was observed. Only one study (10%) was conducted using a popular mean of communication: television (Chart 1).

**Chart 1:** Nursing health technologies in the prevention and control of Hepatitis A – Divinópolis, MG, Brazil, 2024.

Title – author(s) – year – city/country of origin		Objectives:	Design	Participants	Scenario - Level of health care	Technologies for prevention and control of Hepatitis A	Technology classification	Contributions to practice
**E1**	Nurses’ use of the media to provide public health information during a hepatitis A outbreak Davidson and George^(17)^ Moon Township, Pennsylvania, USA 2004	Report the experience carried out	Experience Report	TV Viewers	Media: television	- Health education carried out on television by three nurses on HEP A: transmission, incubation period, hand washing, signs and symptoms, action in case of symptoms, differences between vaccine and immunoglobulin.	Soft-hard	- The opportunity not only highlighted the university and the professionals involved, but demonstrated the scientific-informative nature of nurses on aspects of public health
**E2**	Viral hepatitis prevention education: What do people and providers need to know? Gilbert et al.^(18)^ Atlanta, Georgia, USA. 2005	- Develop a consensus list of key concepts for educational materials on HEP A, B and C for patients and healthcare professionals; - Check what the general population should know about viral hepatitis; - Check what healthcare professionals should know about viral hepatitis; - Produce a list of essential concepts for the general population and health professionals; - Compare recommendations for the general population and healthcare professionals.	Qualitative study, conducted with three populations: experts in viral hepatitis, health professionals and the general population	11 experts in viral hepatitis; 10 health professionals, of which only one was a nurse; and 158 individuals outside the aforementioned groups, aged between 18-49, mostly women	Primary Health Care Tertiary Health Care	- Preparation of questions about viral HEP, from the perspective of experts, health professionals and the general population, with the purpose of verifying essential key concepts in the knowledge related to each group observed; - Formulation of a checklist to guide health education on viral HEP.	Soft-hard	- The study promoted the information that key concepts for the production of informative material must come from different sources, including the target audience;- For future studies, it is suggested that different topics related to health communication be included, such as the scope and reliability of sources, and that sociodemographic criteria, such as ethnicity/race and sexual orientation, be considered when providing health education.
**E3**	Hepatitis in primary care: what NPs can do to save lives Stonsifer et al.^(19)^ Pennsylvania, USA 2006	Report the care practice carried out	Experience Report	3 experts: a nurse, a professor of medicine, and a researcher in epidemiology	Primary Health Care	- Testing of patients during history taking, especially those with liver enzyme abnormalities; history of injecting drug use; patients transfused before 1992; people deprived of liberty; individuals from Africa, Asia, or Eastern Europe; history of risky sexual contact or with men who have sex with men; - Vaccination against HEP A and B for people with chronic liver disease; increased sexual risk; drug use and injection drugs; travelers to or workers from countries at risk of contracting it; health and public safety workers; daycare workers and sewage workers.	Hard	- Need for vaccination against hepatitis A and B in at-risk populations. - Collaboration of the nurse regarding the initial diagnosis and primary care, health education in relation to hepatitis A and B and patient examinations during the collection of information about their clinical history.
**E4**	Effects of a nurse-managed program on hepatitis A and B vaccine completion among homeless adults Nyamathi et al.^(20)^ Los Angeles, California, USA 2009	- Evaluate the effectiveness of a nurse-led case intervention compared to two standard programs to complete the HEP A and B vaccine schedule (combined - Twinrix vaccine) in homeless adults - Access sociodemographic factors and behavioral risk factors related to the completeness of the vaccination schedule	Prospective, quasi-experimental, randomized study conducted with three groups of homeless adults	865 homeless adults, aged 18-65, able and willing to receive immunization against HEP A and B, in an initial immunization program and follow-up after six months	Primary Health Care	- Health education on HEP A; B, C and HIV: means of transmission; diagnosis; prevention; notions about the vaccine against HEP A and B; vaccination schedule; possible reactions and side effects; in addition to its importance; - Health education on topics such as self-esteem, communication, behavior, drug use, sexual behavior and influences on the completeness of the vaccination schedule against HEP A and B; - Monitoring/tracking; - Testing for HEP A; B, C and HIV. - Vaccination against HEP A and B.	Soft-hard, hard	The program demonstrated significant improvement over a six-month schedule (0, 180 days) of vaccinations against HEP A and B relative to standard control The nursing strategy was fundamental to this increase
**E5**	Integrating Health and Prevention Services in Syringe Access Programs: A Strategy to Address Unmet Needs in a High-Risk Population Burr et al.^(21)^ New Jersey, USA 2014	Describe the implementation of a nurse-led project for health promotion and disease prevention, initially designed with the aim of approaching women enrolled in syringe exchange programs for prenatal care and HIV care.	Cross-sectional study	3,488 injecting drug users, of whom 38% were women, visited the needle exchange program facility	Primary Health Care	- Nurse-led program for STI prevention and control, as well as maternal-fetal health promotion for carriers and individuals at risk of HIV; - STI testing; - Immunization against HEP A and B (*Twinrix*).	Soft-hard, hard	- The study demonstrated that the inclusion of nurse-led prevention and health promotion services in syringe exchange services reaches a large number of injecting drug users and pregnant women; - In addition to promoting health through needle exchange, the inclusion of other services such as HIV and hepatitis testing and treatment has expanded the reach, particularly reaching marginalized populations such as injecting drug users.
**E6**	Nursing case management, peer coaching, and Hepatitis A and B vaccine completion among homeless men recently released on parole: randomized clinical trial Nyamathi et al.^(22)^ Los Angeles, California, USA 2015	- Identify which intervention (*intensive peer coaching* and nurse-guided case intervention; intensive peer coaching; and usual care) is more assertive in completing the HEP A and B vaccination schedule; - Identify predictors of completeness of the HEP A and B vaccination schedule	Randomized controlled clinical trial.	345 individuals on probation, included in drug treatment residences	Secondary Health Care	- Formation of pairs (Peer coaching); - Case management, in sessions with a nurse, for 45 minutes/week, for 8 weeks, based on vaccination completion, adherence to the drug rehabilitation program, health promotion and reduction of sexual risk and substance abuse; - Vaccination against HEP A and B (*Twinrix*) following the 0, 7 and 21-30 day schedule.	Soft, soft-hard, hard	- Completeness of the vaccination schedule against HEP A and B - Assessment of the effectiveness of three interventions carried out by nurses, with similarity between the proposed assistance modalities being checked; - Development of multi-level peer and nurse-led programs to improve receptivity to HEP A and B vaccine - Inclusion of nurses in drug treatment residences
**E7**	Viral Hepatitis: New US Screening Recommendations, Assessment Tools, and Treatments Dan et al.^(23)^ Washington, USA 2015	- Analyze the epidemiology and diagnosis of HEP A, HEP B and HEP C; - Discuss the natural history of chronic HEP B and HEP C; - Describe the US Department of Health and Human Services’ action plan, focusing on the role of nurses in prevention, treatment, and, in the case of HCV, cure	Experience Report	- People with HIV infection; - Anyone who has ever used injectable drugs (even once); - Healthcare professionals, emergency physicians and public safety after needlestick injuries or mucosal exposure to blood contaminated with HEP C; - Children born to mothers who tested positive for HEP C;	Primary Health Care	- Serological screening; - Two-step screening process (antibody test and nucleic acid test).	Hard	- Promotion of health education; - Review of action plans against viral hepatitis; - Emphasis on the importance of the nurse’s role in infection control, development and practices related to the aforementioned action plans.
**E8**	Hepatitis A takes hold in the community Heavey^(24)^ Brockport, New York, USA. 2020	To describe HEP A virus infections in at-risk individuals, such as homeless people, in the United States.	Experience Report	General population	Primary Health Care	- Health education on hand washing, hygiene of chlorinated surfaces, adequate consumption of food and water; - Vaccination against HEP A (*Havrix and Vaqta*) and against HEP A and B (*Twinrix*); - Notification to epidemiological surveillance and public health systems; - Consultation for travelers; - Testing for travelers.	Soft-hard, hard	The nurse has an educational and assistance role, especially with immunization, in addition to acting in the prevention and diagnosis of HEP A.
**E9**	Emergency Department-based Hepatitis A Vaccination Program in Response to an Outbreak Kaigh et al.^(25)^ Philadelphia, USA 2020	To assess the incidence of new cases of HEP A after the implementation of a vaccination program guided by best practice advisory (BPA)	Retrospective descriptive cross-sectional study	Data from 5009 individuals visiting an emergency department, perceived as at-risk population (homeless people, drug users, men who have sex with men, recently incarcerated people)	Primary Health Care Secondary Health Care	- Vaccination against HEP A; - Use of BPA, a tool that triggers a reminder, based on each patient’s medical records and history, aiming at checking their vaccination status and eligibility.	Hard	The use of the technologies employed, as well as the association between the public health and emergency departments contributed to the immunization of 669 individuals, and a decrease in the occurrence of HEP A was also observed.
**E10**	An electronic medical record-based intervention to improve hepatitis A vaccination rates in the emergency department during a regional outbreak Bukhsh et al.^(26)^ Michigan, USA 2022	To determine the success of implementing BPA to increase the HEP A vaccination rate in a hospital emergency department, as well as to quantitatively evaluate the use and barriers of the implementation.	Retrospective cohort study and survey analysis.	11,016 patients who presented to an emergency department, screened and verified as individuals at risk for HEP A (homeless people, drug users, incarcerated people, patients with liver disease and MSM)	Secondary Health Care	- BPA encouraging vaccination of individuals at risk for HEP A; - Vaccination against HEP A; - Serological testing.	Hard	The tool used is effective for reminders of HEP A vaccination, but lacks feedback from users (doctors, caregivers and nurses) for future improvements. A massive increase in the number of vaccine requests was observed during the period in which the tool was used.

Note: USA United States of America; HEP: Hepatitis; HIV: human immunodeficiency virus; STI: sexually transmitted infections; BPA: best practice advisory.

**Figure 2:**
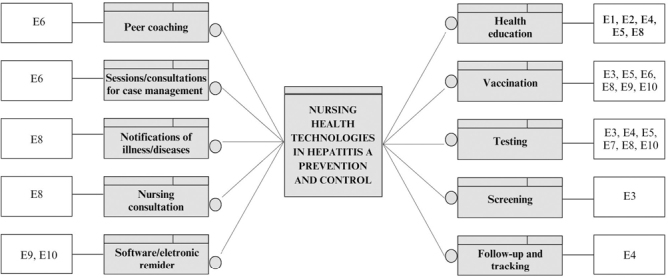
Nursing health technologies in the prevention and control of Hepatitis A – Divinópolis, MG, Brazil, 2024.

Regarding the technologies analyzed, a diverse group was formed, considering the different areas of activity of nursing professionals, namely: vaccination^(19,20,21,24,25,26)^; serological testing^(19,20,21,23,24,26)^; health education^(17,18,20,21,24)^; use of reminders in health information systems^(25,26)^; formation of peer coaching in chronic drug use treatment homes^(22)^; case management^(22)^; consultations^(24)^; notification of diseases and injuries^(24)^; clinical screening^(19)^; and monitoring and tracking^(20)^ (Figure 2).

Regarding the classification of technologies, among the hard technologies, vaccination, testing, clinical screening, and use of Best Practice Advisory (BPA), an electronic reminder in health information systems, were highlighted. The verified soft-hard technologies permeated health education; production of a checklist to guide the health education process; general and at-risk population monitoring and screening; case management carried out by nursing professionals; notification to epidemiological surveillance systems; and consultations for groups prone to contracting the disease. Therefore, soft technologies, due to their social and relational characterization, denoted the formation of peer coaching, described as mutually supportive pairs (Chart 1).

## DISCUSSION

The technologies available in the literature, which nursing professionals have, due to legal and practical competence, are covered in the prevention and control of hepatitis A, in all areas of health care. Heterogeneity was observed in the technologies used, as well as in their use at different levels of health care, highlighting the predominant use of technologies classified as hard.

Among the hard technologies, vaccination stood out with greater recurrence and use. Two types of immunizers used have been described: the combined adsorbed vaccine against hepatitis A and B (*Twinrix* ^©^), and inactivated adsorbed hepatitis A vaccines (*Vaqta* ^©^ and *Havrix* ^©^), recommended and made available by the Advisory Council on Immunization Practices, of the United States Centers for Disease Control and Prevention^(24,27)^. For Soares et al.^(28)^, nursing has knowledge about storage, distribution, administration, adverse effects, and waste disposal measures, playing a leading role and becoming indispensable throughout the vaccination process.

Immunization with the vaccine *Twinrix* ^©^ showed to be advantageous in relation to other immunizers used, given the maintenance of the standard six-month schedule, in which the initial dose and two boosters, 30 and 180 days after the first dose, are administered. Furthermore, immunization against hepatitis A and B with just one vaccine is also noteworthy^(20)^. Additionally, Nyamathi et al.^(22)^ describe the possibility of shortening the vaccination schedule, still with three administrations of the vaccine, with boosters after 7 and between 21 and 30 days after the initial dose. *Havrix* ^©^ and *Vaqta* ^©^, inactivated vaccines, are equivalent to each other, and are recommended depending on the age group, and also following a specific schedule for each age group^(24,27)^.

In reference to vaccination recommendations, in the context analyzed, the inclusion of target groups stood out, in addition to mass vaccination indications for specific age groups. Among the populations, we can mention homeless people; individuals with restricted freedom; LGBTQIAPN+ population, especially MSM and transgender women; injecting drug users; sex workers; travelers to risk areas; sewage workers; and daycare workers^(19,22,24,29)^. It should be noted that, in the Brazilian scenario, the hepatitis A vaccine is made available by the Brazilian Public Health System (SUS), focusing on the target population of children between 15 months and five years old, in a single dose^(30)^; specific groups are also immunized, such as immunocompromised individuals, chronic hepatitis B patients, people living with HIV or AIDS and other specific conditions, based on referral to the Reference Centers for Special Immunobiologicals^(31,32)^.

The influence of a phenomenon known as vaccine hesitancy, or the set of variables that leads to delays and vaccine refusal, in vaccination against hepatitis A is also highlighted, given low coverage rates and epidemics of the disease. Magyar et al.^(33)^ describe, in the Austrian context, parental vaccine hesitancy among children between zero and two years of age, observing greater refusal of the hepatitis A vaccine in relation to the others. For the study, economic factors stand out, especially considering the need to financially acquire the vaccine, and logistical and ideological reasons, tending to validate the occurrences of infections by the hepatitis A virus.

From this perspective, still referring to hard technologies, the relevance of serological testing, or serological screening, was verified for hepatitis A and other STIs, focusing on key populations^(19,20,21,23,24,26)^. Although there is a significant paucity in the literature on hepatitis A serological screening, Bukhsh et al.^(26)^ describe the process of mandatory testing in an emergency department for individuals with increased susceptibility to infection by the virus, in addition to proposing the creation of an electronic tool for reminders about immunization.

Electronic tools, as described by Kaigh et al.^(25)^ and Bukhsh et al.^(26)^, act through reminders in health information systems, based on prompts, or automatic mechanisms resulting from an initial feed to the system, verifying the vaccination indication. The BPA cross-references serological data with sociodemographic variables, articulating the relevance of administering the vaccine, if the individual is indicated, as well as providing information on booster doses, if necessary^(25,26)^.

Additionally, the study conducted by Ford et al.^(34)^ demonstrates the use of BPA for serological screening, verifying the occurrence of individuals infected by the hepatitis C virus, as well as the viral load, aiming at assertiveness in testing patients in an emergency department. The association, therefore, between the use of BPA to screen populations at risk of contracting the hepatitis A virus and vaccination indicated based on serological status, favors transmission control, initial immunization and verification of vaccination completeness, integrating a suitable tool for managing the disease in all health areas.

Since the diagnosis of viral hepatitis is made through testing, individuals included in groups with increased risk or who present suggestive symptoms require serological screening. Verification of specific immunoglobulins for hepatitis A enables not only accurate diagnosis (IgM), but also acquired immunization (IgG), whether through vaccination or previous infection, nevertheless supporting the vaccination indication^(24)^.

Similar to vaccination and testing, the health education process characterized as soft-hard technology, due to the aggregation of knowledge and executions is part of the routine of nursing professionals, constituting an important tool for health promotion and prevention, through guidance, clarification of doubts, and also, adaptation of lifestyles and habits to the health condition^(35)^.

This technology is perceived by the massive influence on the means of transmissive and preventive control of hepatitis A. Different methods and scenarios for educational promotion are described, covering the different groups prone to the pathology. The use of social media at the time television as a means of communication about methods of contagion, immunization, symptoms, behavior, and existing treatments for hepatitis A became evident. The use of mass communication media, such as television, promoting the topic in a popular and targeted way, proved to be not exclusively relevant to clarifying the virus and possible repercussions, but also making explicit the leading role of nursing professionals as holders of knowledge and qualified to administer this information^(17)^.

Meeting the needs of both the academic and general population simultaneously, Gilbert et al.^(18)^ discuss the prospecting of key concepts, essential to the experience of groups of health professionals, care promoters, and citizens, related to hepatitis A. To this end, the groups were stratified, and they were questioned about the concepts considered to be of greatest importance, attached to a group of experts in the illness. The slight lack of preparation of the assistance level regarding certain concepts, which the population demonstrated to be indispensable, was clarified, and subsequently a proposal was made for a checklist for the production of educational material^(18)^.

In observing the diversity of individual and collective educational practices, Nyamathi et al.^(20)^ described the impact of expository sessions, given by nurses, on hepatitis A and other STIs, in addition to adverse effects of vaccination and vaccination frequency in an immunization program for homeless people. In addition to the sessions on prevention and health promotion, behavioral and psychosocial themes proved to be influential in the acceptance and completeness of the proposed vaccination schedule^(20)^, again showing the relevance of the nursing professional in educational and immunization activities.

Individual case management conducted by nursing professionals and associated with educational practices, classified as soft-hard technology, has proven beneficial in promoting health behaviors, such as vaccination and regularization of the vaccination schedule, serological testing, adoption of safe sexual practices and adherence to drug use treatment programs^(22)^. In this context, the educational capacity of nursing is demonstrated, with individual or massive scope. In addition to individual case management, the training of peer coaching, considered as mutually supportive pairs, proved to be of immeasurable value in a program to control chronic drug use, which aimed to verify the propensity for complete vaccination against hepatitis A^(22)^.

Based on the technologies identified, the incipience of national literary production is evident, highlighting the scarcity of approaches aimed at target populations, such as the LGBTQIAPN+ community, workers exposed to risks and homeless people, as observed internationally. Initiatives are therefore proposed for the mass immunization of these vulnerable groups, and the need to develop a scientific and economic framework is evidenced, especially within the scope of Primary Health Care, aiming at the creation of both targeted and general educational campaigns. For the praxis in the nursing clinic, actions such as tracking and serological screening of individuals at imminent risk of infection, checking of current immunization and appropriate treatment based on diagnosis should be part of the essential measures for the control and prevention of hepatitis A.

Possible limitations regarding the research were perceived, such as the scarcity of samples on the aforementioned topic, restricting the analysis and compromising the results; the diversity of methodologies, making the analysis and interpretation of data difficult, which generates a dependence on complementary materials and gray literature, reducing access to unpublished information, thus confirming the need for future investigations in order to strengthen and expand the results.

The lack of knowledge about the legal and practical skills of nursing professionals in addressing hepatitis A, especially in the national scenario, encourages the exploration of the application and documentation of health technologies linked to the disease. Literary productions on the subject not only favor the adoption of care models and scientifically based actions, but also attest to their legality during the exercise and use by nursing professionals.

Mapping health technologies offers a wide range of benefits: to the group of nursing professionals, through the scientific dissemination of practices for adoption and application; to the academic community, through the verification of existing literature, raising hypotheses and conclusions on the subject; to the general population, through the improvement of care actions related to the disease. In this way, the study conducted meets the needs of the aforementioned populations, promoting a concise view of the technologies verified.

## CONCLUSION

This study highlights interventions aimed at preventing and controlling hepatitis A, with an emphasis on different levels of health care. Primary Care stands out as the main focus, mainly addressing vaccination and health education. Secondary and Tertiary Care focus on clinical screening and continuity of care, including the implementation of electronic reminders and systematized protocols for testing and monitoring vaccination in institutionalized patients. In this context, it is important to highlight the role of health technologies used by nursing, which involve both soft technologies (such as support and promotion of interactions) and soft-hard and hard technologies (such as vaccination and electronic reminders).

The concentration of studies of American origin reinforces the perception of literary insufficiency, contrasting health systems and Primary Health Care capable of covering demands for screening, prevention, and treatment of the disease in their responsibilities such as the SUS and the possible negligence and lack of technical preparation in the prevention and control of Hepatitis A.

Regarding sociodemographic aspects, the study highlights strategies aimed at the LGBTQIAPN+ public, homeless people and workers exposed to risk or vulnerability conditions. Finally, in the cultural sphere, educational campaigns are emphasized with the aim of raising awareness among the population about safe sexual practices and preventive measures. This integrated approach aims to improve the coverage and effectiveness of health actions, promoting more accessible and effective care for key populations.

## Data Availability

Data is available on the OSF: https://doi.org/10.17605/OSF.IO/MJESK.

## References

[1] Pereira FEL, Gonçalves CS (2003). Hepatitis A. Rev Soc Bras Med Trop.

[2] Brasil. Ministério da Saúde (2024). Hepatite A.

[3] Brasil. Ministério da Saúde (2018). Manual técnico para diagnóstico das hepatites virais.

[4] Gaspar AMC, Vitral CLA, Oliveira JM, Foccacia R (2013). Tratado de hepatites virais e doenças associadas.

[5] Nainan OV, Xia G, Vaughan G, Margolis HS (2006). Diagnosis of hepatitis A virus infection: a molecular approach. Clin Microbiol Rev.

[6] Migueres M, Lhomme S, Izopet J (2021). Hepatitis A. Epidemiology, high-risk groups, prevention and research on antiviral treatment. Viruses.

[7] Bogdani’c N, Begovac J, Mocibob L, Zekan S, Grgi’c I, Ujevi’c J (2023). Hepatitis A outbreak in men who have sex with men using pre-exposure prophylaxis and people living with HIV in Croatia, January to October 2022. Viruses.

[8] Brasil. Ministério da Saúde (2024). Luta contra Hepatites Virais: Ministério da Saúde lança campanha de conscientização e novo boletim epidemiológico.

[9] Brasil (2024). Ministério da Saúde [Internet]. Instrução Normativa - Calendário Nacional de Vacinação 2024.

[10] Mello VM, Bianchi LM, Sousa PSF, Tavares OS, Di Salvo DRG, Ginuino CF (2022). Increase in Hepatits A Case Linked to Imported Strains to Rio de Janeiro, Brasil: a cross-sectional study. Viruses.

[11] Silveira PO, Guasselli LA, Oliveira GG, Nascimento VF (2021). Relationship between cases of hepatitis A and flood areas, municipality of Encantado, Rio Grande do Sul, Brazil. Cien Saude Colet.

[12] Teles SA (2017). Viral hepatitis: a challenge for nursing. Rev Bras Enferm.

[13] Peters MDJ, Godfrey C, McInerney P, Munn Z, Tricco AC, Khalil H, Aromataris E, Munn Z (2020). Chapter 11: Scoping Reviews (2020 version).

[14] Page MJ, McKenzie JE, Bossuyt PM, Boutron I, Hoffmann TC, Mulrow CD (2022). A declaração PRISMA 2020: diretriz atualizada para relatar revisões sistemáticas. Rev Panam Salud Publica.

[15] Pollock D, Peters MDJ, Khalil H, McInerney P, Alexander L, Tricco AC (2023). Recommendations for the extraction, analysis, and presentation of results in scoping reviews. JBI Evidence Synthesis.

[16] Merhy EE (2002). Saúde: cartografia do trabalho vivo em ato.

[17] Davidson LJ, George LE (2004). Nurses’ use of media to provide public health information during a Hepatitis A outbreak. J Prof Nurs.

[18] Gilbert LK, Bulger J, Scanlon K, Moyer L (2005). Viral hepatitis prevention education: what do people and providers need to know?. Patient Educ Couns.

[19] Stonsifer E, Burke A, Simwale O (2006). Hepatitis in Primary Care: what NPs can do to save lives. Nurse Pract.

[20] Nyamathi A, Liu Y, Marfisee M, Shoptaw S, Gregerson P, Saab S (2009). Effects of a Nurse-Managed Program on Hepatitis A and B vaccine completion among homeless adults. Nurs Res.

[21] Burr CK, Storm DS, Hoyt MJ, Dutton L, Berenzy L, Allread V (2014). Integrating Health and Prevention Services in Syringe Access Programs: A Strategy to Address Unmet Needs in a High-Risk Population. Public Health Rep.

[22] Nyamathi A, Salem BE, Zhang S, Farabee D, Hall B, Kalilifard F (2015). Nursing case management, peer coaching,and Hepatitis A and B vaccine completion among homeless men recently released on parole: randomized clinical trial. Nurs Res.

[23] Dan C, Moses-Eisenstein M, Valdiserri RO (2015). Viral Hepatitis: New U.S. Screening Recommendations, Assessment Tools, and Treatments. Am J Nurs.

[24] Heavey E (2020). Hepatitis A takes hold in the community. Nursing.

[25] Kaigh C, Blome A, Schreyer KE, Healy M (2020). Emergency Department-based Hepatitis A Vaccination Program in Response to an Outbreak. West J Emerg Med.

[26] Bukhsh M, Thyagarajan R, Todd B, Chen NM, Qu L, Swaminatha L (2022). An electronic medical record-based intervention to improve hepatitis A vaccination rates in the emergency department during a regional outbreak. BMJ Open Qual.

[27] Horn EK, Herrera-Restrepo O, Acosta AM, Simon A, Jackson B, Lucas E (2024). The Burden of Hepatitis A Outbreaks in the United States: health outcomes, economic costs, and management strategies. J Infect Dis.

[28] Soares SSS, Souza NVDO, Varella TCMM, Andrade KBS, Pereira SRM, Carvalho EC (2022). The leading role of Nursing in the vaccination against COVID-19 versus questionable irregularities – a descriptive-exploratory study. Online Braz J Nurs.

[29] Osuna MCG, Fuentes MB, Navajo BJ, Porras-Povedano M (2022). Study of the prevention and control campaign for the hepatitis A outbreak in men who have sex with men in Seville (2016-2018). Vacunas.

[30] Brito WI, Souto FJD (2020). Universal hepatitis A vaccination in Brazil: analysis of vaccination coverage and incidence five years after program implementation. Rev Bras Epidemiol.

[31] Brasil. Ministério da Saúde (2023). Manual dos Centros de Referência para Imunobiológicos Especiais.

[32] Brasil. Ministério da Saúde (2024). Manual de Normas e Procedimentos para Vacinação.

[33] Magyar R, Voitl PK, Voitl JJM, Diesner-Treiber S (2024). Vaccine hesitancy among parents of children in their first two years of life. Front Public Health.

[34] Ford JS, Chechi T, Toosi K, Mahmood B, Meehleis D, Otmar M (2021). Universal screening for hepatitis C virus in ED using a Best Practice Advisory. West J Emerg Med.

[35] Costa DA, Cabral KB, Teixeira CC, Mendes JLL, Rosa RR, Cabral FD (2020). Enfermagem e a Educação em Saúde. Rev Cient Esc Estadual Saúde Pública Goiás Cândido Santiago.

