# Supplementary material for: Nursing health technologies in the prevention and control of hepatitis A: a scoping review
Source: Rev Esc Enferm USP. 2025 May 12;59:e20250051. doi: 10.1590/1980-220X-REEUSP-2025-0051en (PMC12080370; doi:10.1590/1980-220X-REEUSP-2025-0051en)
Supplement: Translation

# Translation: Tecnologias em saúde da enfermagem na prevenção e controle da hepatite A: revisão de escopo

**DOI:** 10.1590/1980-220X-REEUSP-2025-0051pt

**Authors:** Leonardo Cassimiro Fonseca e Souza, Camila Stela Gomes, Laís Oliveira de Moraes Tavares, João Pedro Vasconcelos Paolinelli, João Marcos Alves Melo, Valéria Conceição de Oliveira, Brener Santos Silva, Gabriela Gonçalves Amaral

**Affiliations:** 1 Universidade do Estado de Minas Gerais, Divinópolis, MG, Brasil.; 2 Universidade Federal de São João del-Rei, Divinópolis, MG, Brasil.; 3 Universidade do Estado de Minas Gerais, Passos, MG, Brasil.; 4 Universidade de São Paulo, São Paulo, SP, Brasil.

**Keywords:** Hepatite Viral Humana, Hepatite A, Prevenção de Doenças, Controle de Doenças Transmissíveis, Enfermagem

## Abstract

**Objetivo:**

Mapear evidências científicas sobre as tecnologias em saúde da enfermagem na prevenção e controle da Hepatite A.

**Método:**

Revisão de escopo conduzida em 2024 de acordo com as recomendações do JBI e relatada seguindo os critérios do PRISMA-ScR. Realizou-se a busca (10/06/2024) em bases de dados e literatura cinzenta. Classificaram-se as tecnologias encontradas em: leves, leve-duras e duras. Os resultados foram analisados descritivamente e sintetizados.

**Resultados:**

Foram selecionados 10 estudos, todos originados dos EUA. Dentre as tecnologias estão a vacinação; testagem sorológica; educação em saúde; lembretes em sistemas de informação em saúde; formação de *peer coaching*; gestão de caso; consultas; notificação de doenças e agravos; triagem clínica; e acompanhamento e rastreio. Prevaleceram as tecnologias duras.

**Conclusão:**

A Atenção Primária à Saúde destacou-se como o principal foco, abordando principalmente a vacinação e a educação em saúde. Já a Atenção Secundária e a Terciária concentraram-se na triagem clínica e na continuidade do cuidado. A concentração dos estudos de origem estadunidense reforça a percepção de insipiência literária e a possível negligência e falta de preparo técnico na prevenção e controle da Hepatite A.

## INTRODUÇÃO

A hepatite A é causada pelo vírus A, que pertence à família *Picornaviridae* e ao gênero *Hepatovirus*, e é frequentemente chamada de “hepatite infecciosa”^(1)^. Os sintomas, quando presentes, são geralmente inespecíficos, começando com fadiga, mal-estar, febre e dores musculares, seguidos por manifestações gastrointestinais como enjoo, vômitos, dor abdominal, constipação ou diarreia. A urina escura pode aparecer antes da icterícia. Os sintomas costumam surgir de 15 a 50 dias após a infecção e tendem a durar menos de dois meses^(2)^.

Durante a fase aguda, ocorre a replicação viral inicial, acompanhada pela eliminação do vírus nas fezes. A transmissibilidade pode ocorrer a partir de duas semanas antes até pelo menos uma semana após o início da icterícia ou de outros sintomas clínicos, além da elevação das enzimas hepáticas. O vírus pode ser detectado nas fezes cerca de uma a duas semanas após a exposição, persistindo, em média, por 79 dias após o pico da infecção^(3,4,5)^.

A infecção em indivíduos com mais de 50 anos tende a evoluir de forma mais grave e sintomática, com icterícia em mais de 70% dos pacientes. O período de incubação é, em média, de 28 dias, variando de 15 a 50 dias. Embora não haja relatos de hepatite A levando a hepatite crônica ou hepatocarcinoma, podem ocorrer casos de hepatite fulminante^(4,5)^. Raramente, há presença de complicações, sendo a mais grave a insuficiência cardíaca fulminante, que ocorre em 0,5% dos casos, além de manifestações extra-hepáticas, como hemólise, colestase acalculosa, derrames pleurais e pericárdicos, artrite reativa, pancreatite e manifestações neurológicas^(1)^.

A transmissão da hepatite A se dá principalmente pela via oral-fecal, associada ao consumo de alimentos ou água contaminados, e é influenciada por baixos níveis de saneamento básico e higiene pessoal. Outras formas de transmissão incluem o contato pessoal próximo (como entre moradores da mesma casa ou crianças em creches), além de contato sexual, especialmente por vias oro-anal ou dígito-anal^(4)^. Entre as populações com maior risco de contração por via sexual, homens que fazem sexo com homens (HSH) são um grupo que contribui para o aumento da incidência, incluindo homens gays, bissexuais, não-binários e heterossexuais que se relacionam sexualmente com outros homens^(6)^.

Mundialmente, há um aumento significativo na ocorrência de hepatite A aguda transmitida por via sexual, especialmente entre HSH. Na União Europeia, entre 2016 e 2018, foram documentados 19.947 casos, o que representa um aumento de quatro vezes em comparação com o triênio anterior (2012 a 2015)^(7)^. No Brasil, de 2000 a 2021, foram notificados 718.651 casos confirmados de hepatites virais, dos quais 168.175 (23,4%) eram de hepatite A. Apesar da redução geral nas notificações, que alcançou 95,6% entre 2011 e 2021^(8)^, essa queda deve-se em grande parte à ampla cobertura vacinal oferecida gratuitamente para crianças até 4 anos, 11 meses e 29 dias^(9)^. No entanto, em regiões específicas, como o estado do Rio de Janeiro, observou-se um aumento significativo em 2018, com 9 casos por 100.000 habitantes, em contraste com 0,3 casos por 100.000 habitantes no ano anterior. Esse aumento foi especialmente notável entre a população de HSH^(10)^.

Complementarmente, a natureza de transmissão da hepatite A é influenciada por desastres naturais que afetam a distribuição de alimentos e água. A recorrência de enchentes, como as ocorridas no Rio Grande do Sul em maio de 2024, favorece a disseminação da doença, evidenciando fatores sociodemográficos que contribuem para a propagação^(11)^. Isso ressalta a urgência de implementar medidas eficazes de controle e prevenção.

O cuidado de enfermagem é fundamental em todas as ações de controle e prevenção de infecções, abrangendo diversas áreas. Isso inclui o sistema de vigilância das hepatites virais, que monitora as tendências da doença; o planejamento de estratégias de prevenção e controle; a triagem clínica de potenciais doadores de sangue e derivados nos bancos de sangue; a administração de vacinas e campanhas de imunização; a assistência às gestantes para prevenir a transmissão vertical; o rastreamento de casos de hepatites virais; a assistência terciária a indivíduos com formas agudas e crônicas das infecções; e a produção de conhecimentos que possibilitem a realização segura de práticas baseadas em evidências^(12)^.

É importante ressaltar que a hepatite A é menos abordada em comparação com outras infecções, como hepatite B, sífilis e HIV, entre outras infecções sexualmente transmissíveis (IST). Isso resulta em carência significativa de conhecimento técnico-prático e na sistematização da assistência aos pacientes acometidos com essa infecção. Além disso, há falta de protagonismo e participação da enfermagem na abordagem da hepatite A, especialmente considerando sua transmissão sexual e as especificidades de morbimortalidade. Portanto, há necessidade de envolvimento dos profissionais de enfermagem na prevenção e promoção da saúde relacionada à hepatite A.

Assim, este estudo tem como objetivo mapear evidências científicas sobre as tecnologias em saúde da enfermagem na prevenção e controle da hepatite A.

## MÉTODO

### Desenho do Estudo

Trata-se de uma revisão de escopo conduzida de acordo com as recomendações do *Joanna Briggs Institute* (JBI) – *Manual for Evidence Synthesis for Scoping review* ^(13)^; e relatada seguindo os critérios do *Preferred Reporting Items for Systematic reviews and Meta-Analyses extension for Scoping Reviews* (PRISMA-ScR)^(14)^. O protocolo da revisão está registrado no *Open Science Framework* (OSF) *Registries* (https://doi.org/10.17605/OSF.IO/MJESK).

### Fontes de Dados e Estratégia de Pesquisa

Foi realizada uma busca preliminar, em maio de 2024, nas bases de dados OSF e PROSPERO, do *National Institute for Health and Care Research*, para mapear e verificar estudos semelhantes em andamento ou concluídos, avaliando a abrangência e a metodologia utilizadas, sem encontrar registros relevantes.

Para definir a questão norteadora, utilizou-se o mnemônico População-Conceito-Contexto (PCC). Considerou-se como população (P) os profissionais de enfermagem; como conceito (C), as tecnologias empregadas na prevenção e controle da hepatite A; e como contexto (C) todos os níveis de atenção à saúde. Consideraram-se tecnologias as diferentes habilidades técnico-práticas utilizadas por profissionais enfermeiros durante a assistência, sejam estas referentes à clínica, educação, vigilância epidemiológica, prevenção e promoção da saúde ou, ainda, tecnologias físicas, materiais. Desta forma, a questão norteadora formulada foi: “Quais são as tecnologias em saúde utilizadas pelos profissionais de enfermagem na prevenção e controle da hepatite A?”

Foram realizadas buscas no Portal da Biblioteca Virtual em Saúde (BVS), sendo incluídas as bases *National Library of Medicine’s* (NLM – MEDLINE)/*National Library of Medicine and National Institutes of Health* (PubMed); *Latin American and Caribbean Health Sciences* (LILACS); Índice Bibliográfico Español en Ciencias de la Salud (IBECS); e Base de dados de Enfermagem (BDEnf). Ademais, foram verificadas as bases PubMed/Medline; EMBASE; Scopus; *Web of Science* (WOS); CINAHL *Database;* e *Cochrane Library*. Para a literatura cinzenta, foram consultados o Catálogo de Teses e Dissertações da Coordenação de Aperfeiçoamento de Pessoal de Nível Superior (CAPES); a *Theses Canada;* e *World Cat Dissertations and Theses*. Por fim, foram verificadas as referências dos estudos analisados para buscar literatura complementar.

A busca foi dividida em etapas: a) busca preliminar para verificar estudos similares em andamento, juntamente com a identificação de termos de pesquisa e palavras-chave; b) a reunião de termos e palavras-chave, seguida da formulação das estratégias de busca e sua adequação às especificidades das bases consultadas; c) a verificação dos resultados e a seleção dos registros utilizados. As estratégias de busca, ajustadas para cada base, estão publicadas como material suplementar, disponível em https://doi.org/10.17605/OSF.IO/MJESK.

Foi definida a inclusão de artigos originais com abordagens quantitativas, qualitativas ou mistas; disponíveis integralmente; sem restrição temporal; nos idiomas português, inglês e espanhol; que abordassem tecnologias utilizadas por profissionais de enfermagem na prevenção e controle da hepatite A. Também foram incluídos relatos de experiência. Foram excluídas revisões de literatura, protocolos de estudo, cartas, editoriais e pôsteres. Além disso, foram descartados estudos de caráter puramente epidemiológico, estudos não relacionados à população humana e ou que tratassem de produtos alimentícios.

### Coleta e Extração dos Dados

Após a consulta às bases de dados, realizada em 10/06/2024, os registros obtidos foram importados para o *software Rayyan*, que auxilia na seleção, começando pela remoção de duplicatas. Dois revisores independentes realizaram a triagem por título e resumo. Com base nessa seleção, os textos foram analisados na íntegra para verificar a pertinência e o cumprimento dos critérios de elegibilidade, por dois revisores independentes, seguindo-se o método de revisão duplo-cego. Em caso de impasses ou dúvidas, um terceiro revisor foi consultado. Dos registros excluídos pela indisponibilidade integral, realizou-se tentativa de contato com a autoria dos estudos, via *e-mail*, para solicitação da obra, contudo, sem retorno. O diagrama de fluxo PRISMA-ScR^(14)^ foi utilizado para relatar os resultados de busca e registrar os motivos dos artigos excluídos.

Foi utilizado um instrumento de coleta de dados desenvolvido pelos autores, que foi avaliado por meio de um teste- piloto com a extração de dados de cinco artigos para calibração. As variáveis coletadas incluíram: título, autor(es), país, ano de publicação, objetivo(s), delineamento do estudo, participantes, cenário do estudo, nível de assistência à saúde, tecnologias para prevenção e controle da hepatite A, classificação da tecnologia, além de conclusões e recomendações. Coerente com as recomendações da JBI^(15)^, não foi realizada a análise individual do risco de viés dos estudos selecionados.

Para classificação das tecnologias seguiu-se o referencial teórico descrito por Merhy^(16)^, sendo divididas em tecnologias leves, que indicam aquelas referentes às relações humanas, como a produção de vínculo e das relações, autonomização, acolhimento, gestão de processos de trabalho; leve-duras, como no caso dos saberes bem estruturados, que operam no trabalho em saúde, como a clínica médica, a psicanalítica, a epidemiológica, entre outras; e duras, como no caso de equipamentos tecnológicos, máquinas, normas, estruturas organizacionais.

### Síntese dos Resultados

A partir dos dados extraídos, foi elaborado um resumo das evidências, organizado em quadros, juntamente com a interpretação e síntese geral dos achados, destacando lacunas de conhecimento. Também foram levantadas as contribuições das tecnologias disponíveis, além de sugestões para políticas públicas e a evolução da prática clínica de enfermagem.

### Aspectos Éticos

Por tratar-se de uma revisão de escopo, não foi necessário submeter o estudo à apreciação de um Comitê de Ética em Pesquisa.

## RESULTADOS

A partir das buscas nas bases de dados, foram retornados 3.453 registros. Após a exclusão de 815 duplicatas, restaram 2.638 arquivos possivelmente elegíveis para a revisão. A leitura de títulos e resumos foi realizada para verificar os critérios de elegibilidade, resultando na exclusão de 2.608 publicações e deixando 30 estudos para leitura integral. Por fim, foram selecionados 10 estudos. Os detalhes da etapa de busca, identificação e seleção dos estudos estão descritos na Figura 1.

**Figura 1:** Fluxograma de identificação, seleção, elegibilidade e inclusão dos estudos, com base nas recomendações do PRISMA – Divinópolis, MG, Brasil, 2024.

Os estudos foram publicados entre os anos de 2004 e 2022, com maior ocorrência em 2015 (n = 2; 20%) e 2020 (n = 2; 20%). No que tange à nacionalidade, no total, as publicações ocorreram nos Estados Unidos, com destaque para os estados da Pensilvânia (n = 3; 30%) e Califórnia (n = 2; 20%). Quanto ao delineamento dos estudos, quatro (40%) tratavam-se de relatos de experiência; dois (20%) estudos transversais; um (10%) estudo quase experimental; um (10%) ensaio clínico randomizado; um (10%) estudo descritivo qualitativo; e um (10%) estudo de coorte retrospectivo. Em relação aos níveis de atenção à saúde, destacou-se a Atenção Primária à Saúde (APS) (n = 5; 50%) e a Atenção Secundária à Saúde (n = 2; 20%). Além disso, foi observada a interseção entre APS e Secundária (n = 1; 10%) e APS e Terciária (n = 1; 10%). Apenas um estudo (10%) foi conduzido tendo como cenário um meio de comunicação popular: a televisão (Quadro 1).

**Quadro 1:** Tecnologias em saúde da enfermagem na prevenção e controle da Hepatite A – Divinópolis, MG, Brasil, 2024.

Título – autor(es) – ano – cidade/país de origem		Objetivo(s)	Delineamento	Participantes	Cenário - Nível de assistência à saúde	Tecnologias para prevenção e controle da Hepatite A	Classificação da tecnologia	Contribuições para a prática
**E1**	Nurses’ use of the media to provide public health information during a hepatitis A outbreak Davidson e George^(17)^ Moon Township, Pensilvânia, EUA 2004	Relatar a experiência realizada	Relato de experiência	Telespectadores	Meio de comunicação: televisão	- Educação em saúde realizada em rede de televisão, por três enfermeiros, sobre HEP A: transmissão, período de incubação, lavagem de mãos, sinais e sintomas, ação em caso de sintomatologia, diferenças sobre vacina e imunoglobulina.	Leve-duras	- A oportunidade não somente promoveu destaque à universidade e aos profissionais envolvidos, mas demonstrou o caráter científico-informativo de enfermeiros sobre aspectos de saúde pública
**E2**	Viral hepatitis prevention education: What do people and providers need to know? Gilbert et al.^(18)^ Atlanta, Georgia, EUA. 2005	- Desenvolver uma lista consensual de conceitos-chave a materiais educativos sobre HEP A, B e C para pacientes e profissionais da saúde;<br/>- Verificar o que a população em geral deve saber sobre hepatites virais;<br/>- Verificar o que profissionais da saúde devem saber sobre hepatites virais;<br/>- Produzir uma lista de conceitos essenciais à população em geral e a profissionais da saúde;<br/>- Comparar as recomendações à população em geral e aos profissionais da saúde.	Estudo qualitativo, conduzido com três populações: *experts* em hepatites virais, profissionais da saúde e população em geral	11 *experts* em hepatites virais;<br/>10 profissionais da saúde, dos quais, apenas um enfermeiro; e 158 indivíduos fora dos grupos supracitados, com idades entre 18-49, majoritariamente mulheres	Atenção Primária à Saúde Atenção Terciária à Saúde	- Elaboração de questões acerca de HEP virais, na perspectiva de *experts*, profissionais da saúde e população em geral, com a finalidade de verificar conceitos-chave essenciais no conhecimento referente à cada grupo observado;<br/>- Formulação de uma checklist para norteio de educação em saúde sobre HEP virais.	Leve-duras	- O estudo promoveu a informação de que os conceitos-chave para a produção de material informativo devem advir de diversas fontes, incluindo o público ao qual se destina;<br/>- Para futuros estudos, é sugerida a inclusão de tópicos diversos relacionados à comunicação em saúde, como alcance e confiabilidade das fontes, bem como devem ser considerados critérios sociodemográficos, como etnia/raça e orientação sexual durante a realização de educação em saúde
**E3**	Hepatitis in primary care: what NPs can do to save lives Stonsifer et al.^(19)^ Pensilvânia, EUA 2006	Relatar a prática assistencial realizada	Relato de experiência	3 experts:<br/>um enfermeiro, um professor de medicina e um pesquisador em epidemiologia	Atenção Primária à Saúde	- Testagem de pacientes durante anamnese, especialmente aqueles com anormalidades de enzimas hepáticas; histórico de uso de drogas injetáveis; transfusionados anteriormente a 1992; pessoas privadas de liberdade; indivíduos provenientes da África, Ásia ou Leste Europeu; histórico de contato sexual de risco ou com homens que fazem sexo com homens;<br/>- Vacinação contra HEP A e B para pessoas com doença hepática crônica; risco sexual aumentado; uso de drogas e drogas injetáveis; viajantes ou trabalhadores de países em risco de contração; trabalhadores da saúde e segurança pública; trabalhadores de creches e trabalhadores do esgoto.	Duras	- Necessidade de vacinação contra hepatite A e B em população de risco. <br/>- Colaboração do enfermeiro acerca do diagnóstico inicial e dos cuidados primários, na educação em saúde em relação às hepatites A e B e os exames dos doentes durante a coleta de informações sobre sua história clínica.
**E4**	Effects of a nurse-managed program on hepatitis A and B vaccine completion among homeless adults Nyamathi et al.^(20)^ Los Angeles, Califórnia, EUA2009	- Avaliar a efetividade de uma intervenção de caso guiada por enfermeiro, comparada a dois programas padrões para completar o esquema vacinal da vacina das HEP A e B (combinadas - vacina Twinrix) em adultos em situação de rua<br/>- Acessar fatores sociodemográficos e fatores comportamentais de risco relacionados à completude do esquema vacinal	Estudo prospectivo, quasi-experimental, randomizado, conduzido com três grupos de adultos em situação de rua	865 adultos em situação de rua, entre 18-65 anos, em condições e aceitação de receber imunização contra HEP A e B, em um programa de imunização inicial e retorno após seis meses	Atenção Primária à Saúde	**-**Educação em saúde sobre HEP A; B, C e HIV: meios de transmissão; diagnóstico; prevenção; noções sobre a vacina contra HEP A e B; esquema vacinal; possíveis reações e efeitos colaterais; além de sua importância;<br/>**-**Educação em saúde sobre temas como autoestima, comunicação, comportamento, uso de drogas, comportamento sexual e influências sobre a completude do esquema vacinal contra HEP A e B;<br/>**-**Acompanhamento/rastreio;<br/>**-**Testagem para HEP A; B, C e HIV.<br/>**-**Vacinação contra HEP A e B.	Leve-duras, duras	O programa demonstrou uma melhora significativa em relação às vacinações de um esquema de seis meses (0, 180 dias) contra HEP A e B em relação ao controle padrão A estratégia de enfermagem foi fundamental para esse aumento
**E5**	Integrating Health and Prevention Services in Syringe Access Programs: A Strategy to Address Unmet Needs in a High-Risk Population Burr et al.^(21)^ New Jersey, EUA 2014	Descrever a implementação de um projeto guiado por enfermeiro para promoção de saúde e prevenção de doenças, inicialmente pensado com o objetivo de abordar mulheres, inseridas em programas de troca de seringa, para realização de pré-natal e cuidados contra o HIV	Estudo transversal	3488 usuários de drogas injetáveis, dos quais, 38% eram mulheres, que visitaram a instalação do programa de troca de seringas	Atenção Primária à Saúde	**-**Programa guiado por enfermeiro para prevenção e controle de IST, bem como promoção de saúde materno-fetal para portadores e indivíduos em risco de contração de HIV;<br/>**-**Testagem contra IST;<br/>**-**Imunização contra HEP A e B (*Twinrix*).	Leve-duras, duras	- O estudo demonstrou que a inclusão de serviços de prevenção e promoção da saúde, guiados por enfermeiro, nos serviços de troca de seringas atinge grande número de usuários de drogas injetáveis e mulheres grávidas;<br/>- Além da promoção da saúde por meio da troca de seringas, a inclusão de outros serviços, como testagem e tratamento de HIV e hepatites expandiu o alcance, sobretudo atingindo populações marginalizadas, como os usuários de drogas injetáveis
**E6**	Nursing case management, peer coaching,and Hepatitis A and B vaccine completion among homeless men recently released on parole: randomized clinical trial Nyamathi et al.^(22)^ Los Angeles, California, EUA 2015	- Identificar qual intervenção (*intensive peer coaching* e intervenção de caso guiada por enfermeiro; *intensive peer coaching*; e assistência usual) é mais assertiva na completude do esquema vacinal de HEP A e B; - Identificar os preditores da completude do esquema vacinal de HEP A e B	Ensaio clínico randomizado	345 indivíduos em situação de liberdade condicional, incluídas em residências de tratamento contra o uso de drogas	Atenção Secundária à Saúde	- Formação de duplas (*Peer coaching)*; - Gestão do caso, em sessões com enfermeiro, durante 45 minutos/semana, durante 8 semanas, baseada em completude da vacinação, adesão ao programa de reabilitação de drogas, promoção de saúde e diminuição do risco sexual e do abuso de substâncias; - Vacinação contra HEP A e B (*Twinrix*) seguindo o esquema de 0, 7 e 21-30 dias.	Leves, leves-duras, duras	- Completude do esquema vacinal contra o HEP A e B - Avaliação da eficácia de três de intervenções realizadas pelos enfermeiros, sendo verificada similaridade entre as modalidades de assistência propostas; - Desenvolvimento de vários níveis de programas orientados por colegas e enfermeiros para melhor receptividade à vacina contra HEP A e B - Inclusão de enfermeiros nas residências de tratamento contra o uso de drogas
**E7**	Viral Hepatitis: New U.S. Screening Recommendations, Assessment Tools, and Treatments Dan et al.^(23)^ Washington, EUA 2015	- Analisar a epidemiologia e o diagnóstico do HEP A, HEP B e HEP C; - Discutir a história natural do HEP B e HEP C crônicos; - Descrever o plano de ação do Departamento de Saúde e Serviços Humanos dos EUA, com foco no papel dos enfermeiros na prevenção, tratamento e, no caso do VHC, na cura	Relato de experiência	- Pessoas com infecção pelo HIV; - Qualquer pessoa que já tenha usado drogas injetáveis (mesmo que uma vez); - Profissionais de saúde, médicos de emergência e segurança pública após ferimentos por agulha ou exposição da mucosa a sangue contaminado com HEP C; - Crianças nascidas de mães que testaram positivo para o HEP C;	Atenção Primária à Saúde	- Rastreamento sorológico; - Processo de triagem em duas etapas (teste de anticorpos e teste de ácido nucleico).	Duras	- Promoção da educação em saúde; - Revisão dos planos de ação contra hepatites virais; - Enfatizar a importância do papel do enfermeiro no controle de infecções, desenvolvimento e práticas relacionadas aos planos de ação supracitados.
**E8**	Hepatitis A takes hold in the community Heavey^(24)^ Brockport, Nova York, EUA. 2020	Descrever as infecções pelo vírus da HEP A em indivíduos em risco, como pessoas em situação de rua, nos Estados Unidos.	Relato de experiência	População em geral	Atenção Primária à Saúde	- Educação em saúde sobre lavagem de mãos, higiene de superfícies com cloro, consumo adequado de alimentos e água; - Vacinação contra HEP A (*Havrix e Vaqta*) e contra HEP A e B (*Twinrix*); - Notificação aos sistemas de vigilância epidemiológica e saúde pública; - Consulta para viajantes; - Testagem para viajantes.	Leve-duras, duras	O enfermeiro tem papel educador e assistencial, sobretudo com a imunização, além de atuar na prevenção e diagnóstico da HEP A.
**E9**	Emergency Department-based Hepatitis A Vaccination Program in Response to an Outbreak Kaigh et al.^(25)^ Philadelphia, EUA 2020	Avaliar a incidência de novos casos de HEP A após a implementação de um programa de vacinação guiado por *best practice advisory* (BPA)	Estudo transversal descritivo retrospectivo	Dados de 5009 indivíduos visitantes de um setor de emergência, percebidos como população em risco (pessoas em situação de rua, usuários de drogas, homens que fazem sexo com homens, pessoas recentemente encarceradas)	Atenção Primária à Saúde Atenção Secundária à Saúde	- Vacinação contra HEP A; - Uso de BPA, uma ferramenta que dispara um lembrete, com base no prontuário e histórico de cada paciente, com a finalidade de verificar a situação e elegibilidade vacinal.	Duras	O uso das tecnologias empregadas, bem como a associação entre os departamentos de saúde pública e emergência contribuíram para a imunização de 669 indivíduos, sendo também verificada diminuição da ocorrência de HEP A.
**E10**	An electronic medical record-based intervention to improve hepatitis A vaccination rates in the emergency department during a regional outbreakBukhsh et al.^(26)^ Michigan, EUA 2022	Determinar o sucesso da implementação de BPA para aumento da taxa vacinal contra HEP A no departamento de emergência de um hospital, bem como avaliar quantitativamente o uso e barreiras do implementado.	Estudo de coorte retrospectiva e *survey analysis*.	11.016 pacientes que deram entrada em um setor de emergência, triados e verificados como indivíduos em risco para HEP A (pessoas em situação de rua, usuários de drogas, encarcerados, pacientes com doença hepática e HSH)	Atenção Secundária à Saúde	- BPA incitando a vacinação de indivíduos em risco para HEP A; - Vacinação contra HEP A; - Testagem sorológica.	Duras	A ferramenta utilizada é efetiva para lembretes da vacinação contra HEP A, mas carece do feedback dos utilizadores (médicos, cuidadores e enfermeiros) para futuras melhorias. Foi verificado massivo aumento do número de solicitações de vacinas durante o período de uso da ferramenta.

Nota: EUA: Estados Unidos da América; HEP: Hepatite; HIV: vírus da imunodeficiência humana; IST: infecções sexualmente transmissíveis; BPA: *best practice advisory*.

Tocante às tecnologias analisadas, formou-se um grupo diverso, considerando as diferentes áreas de atuação dos profissionais de enfermagem, a saber: vacinação^(19,20,21,24,25,26)^; testagem sorológica^(19,20,21,23,24,26)^; educação em saúde^(17,18,20,21,24)^; uso de lembretes em sistemas de informação em saúde^(25,26)^; formação de *peer coaching* em residências de tratamento contra uso crônico de drogas^(22)^; gestão de caso^(22)^; consultas^(24)^; notificação de doenças e agravos^(24)^; triagem clínica^(19)^; e acompanhamento e rastreio^(20)^ (Figura 2).

**Figura 2:** Tecnologias em saúde da enfermagem na prevenção e controle da hepatite A – Divinópolis, MG, Brasil, 2024.

Acerca da classificação das tecnologias, dentre as tecnologias duras evidenciou-se a vacinação; testagem; triagem clínica; e uso de *Best Practice Advisory* (BPA), um lembrete eletrônico nos sistemas de informação de saúde. As tecnologias leves-duras verificadas permearam a educação em saúde; produção de *checklist* para nortear o processo educativo em saúde; acompanhamento e rastreio populacional geral e de risco; gestão de caso realizada pelo profissional enfermeiro; notificação aos sistemas de vigilância epidemiológica; e consultas para grupos propensos à contração. Por conseguinte, as tecnologias leves, por sua caracterização social e relacional, denotaram a formação de *peer coaching*, descritas como duplas de apoio mútuo (Quadro 1).

## DISCUSSÃO

Foram abrangidas as tecnologias disponíveis na literatura, as quais os profissionais de enfermagem têm por competência legal e prática na prevenção e controle da hepatite A, em todos os âmbitos de assistência à saúde. Observou-se heterogeneidade nas tecnologias utilizadas, bem como em seu emprego nos distintos níveis de atenção à saúde, destacando-se o uso predominante de tecnologias classificadas como duras.

Dentre as tecnologias duras, destacou-se, com maior recorrência e utilização, a vacinação. Foram descritos dois tipos de imunizantes utilizados: a vacina adsorvida combinada contra hepatite A e B (*Twinrix^©^*), e as vacinas adsorvidas inativadas contra hepatite A (*Vaqta^©^* e *Havrix^©^*), recomendadas e disponibilizadas pelo Conselho Consultivo das Práticas de Imunização, do Centro de Controle e Prevenção de Doenças dos Estados Unidos^(24,27)^. Para Soares et al.^(28)^, a enfermagem detém saberes acerca do armazenamento, distribuição, administração, efeitos adversos e medidas de descartes de resíduos, protagonizando e tornando-se indispensável em todo o processo de vacinação.

A imunização com a vacina *Twinrix^©^* demonstra-se vantajosa em relação aos demais imunizantes utilizados, dado o mantenimento do esquema padrão de seis meses, no qual administra-se a dose inicial, e dois reforços, passados 30 e 180 dias da primeira dose. Ademais, destaca-se, também, a imunização contra as hepatites A e B com apenas um imunizante^(20)^. Complementarmente, Nyamathi et al.^(22)^ descrevem a possibilidade de abreviação do esquema vacinal, ainda com três administrações do imunizante, com os reforços após 7 e entre 21 e 30 dias à dose inicial. *Havrix^©^* e *Vaqta^©^*, vacinas inati­vadas, apresentam equivalência entre si, sendo recomendadas a depender da faixa etária, observando-se, também, esquema específico por idade^(24,27)^.

Em referência à recomendação vacinal, no contexto analisado, sobressaiu-se a inclusão de grupos alvos, ademais às indicações vacinais em massa de faixas etárias específicas. Dentre as populações, citam-se pessoas em situação de rua; indivíduos restritos de liberdade; população LGBTQIAPN+, sobretudo HSH e mulheres transsexuais; usuários de drogas injetáveis; profissionais do sexo; viajantes a áreas de risco; trabalhadores do esgoto; e trabalhadores de creches^(19,22,24,29)^. Salienta-se que, no cenário brasileiro, a vacina contra a hepatite A é disponibilizada pelo Sistema Único de Saúde (SUS), enfocando a população-alvo de crianças entre 15 meses e cinco anos, em dose única^(30)^; também são imunizados grupos específicos, como imunodeprimidos, pacientes crônicos de hepatite B, pessoas vivendo com HIV ou AIDS e outras condições pontuais, a partir da referenciação aos Centros de Referência para Imunobiológicos Especiais^(31,32)^.

Também se realça a influência de um fenômeno conhecido como hesitação vacinal, ou o conjunto de variáveis que acarreta em atrasos e recusa vacinal, na vacinação contra hepatite A, tendo em vista baixos índices de cobertura e epidemias da doença. Magyar et al.^(33)^ descrevem, no contexto austríaco, a hesitação vacinal parental de crianças entre zero e dois anos, observando-se maior recusa ao imunizante contra hepatite A em relação aos demais. Para o estudo, destacam-se fatores econômicos, mormente, tendo em vista a necessidade de adquirir financeiramente a vacina, ademais a motivos logísticos e ideológicos, tendendo a validar as ocorrências de infecções pelo vírus da hepatite A.

Nesta perspectiva, ainda em referência às tecnologias duras, foi verificada relevância da testagem sorológica, ou rastreio sorológico, para hepatite A e demais IST, com enfoque em populações chaves^(19,20,21,23,24,26)^. Embora haja escassez significativa na literatura sobre o rastreio sorológico da hepatite A, Bukhsh et al.^(26)^ descrevem o processo de testagem obrigatória em um departamento de emergência para indivíduos com maior susceptibilidade à infecção pelo vírus, além de proporem a criação de uma ferramenta eletrônica para lembretes sobre a imunização.

As ferramentas eletrônicas, como descrito por Kaigh et al.^(25)^ e Bukhsh et al.^(26)^, atuam mediante lembretes nos sistemas de informação em saúde, a partir de *prompts*, ou mecanismos automáticos decorrentes de uma alimentação inicial ao sistema, verificando a indicação vacinal. O BPA realiza o cruzamento de dados sorológicos com variáveis sociodemográficas, articulando a pertinência da administração de imunizante, caso o indivíduo apresente indicação, bem como fornecendo informações sobre doses de reforço, se necessário^(25,26)^.

Complementarmente, o estudo conduzido por Ford et al.^(34)^ demonstra o uso de BPA para rastreio sorológico, verificando a ocorrência de indivíduos infectados pelo vírus da hepatite C, bem como a carga viral, objetivando assertividade na testagem de pacientes em um departamento de emergência. A associação, por conseguinte, entre o uso de BPA para rastreio de populações em risco de contração do vírus da hepatite A e a vacinação indicada a partir do status sorológico, favorece o controle transmissivo, imunização inicial e verificação da completude vacinal, integrando ferramenta propícia para o manejo da doença em todos os âmbitos de saúde.

Sendo o diagnóstico de hepatites virais realizado a partir da testagem, indivíduos incluídos em grupos com risco aumentado ou que apresentem sintomatologia sugestiva necessitam de rastreamento sorológico. A verificação de imunoglobulinas específicas para hepatite A possibilita, não somente, o diagnóstico preciso (IgM), como também a imunização adquirida (IgG), seja por meio de vacinação ou de infecção prévia, apoiando, não obstante, a indicação vacinal^(24)^.

De modo similar à vacinação e testagem, o processo educativo em saúde caracterizado como tecnologia leve-dura, pelo agregamento de saberes e execuções é integrante da rotina dos profissionais de enfermagem, constituindo importante artifício para a promoção e prevenção da saúde, mediante a orientação, esclarecimentos de dúvidas, e ainda, adaptação de estilos e hábitos de vida à condição de saúde^(35)^.

Tal tecnologia é percebida pela massiva influência nos meios de controle transmissivo e preventivo de hepatite A. Ficam descritos diferentes modos e cenários para a promoção educativa, abarcando os diferentes grupos propensos à patologia. Evidenciou-se a utilização das mídias sociais à época a televisão como meio de comunicação acerca de métodos de contágio, imunização, sintomatologia, condutas e tratamentos existentes para hepatite A. O uso do meio comunicativo em massa, como a rede televisiva, promovendo a temática de modo popular e direcionado, demonstrou-se não exclusivamente pertinente ao esclarecimento acerca do vírus e possíveis repercussões, sendo explícito, também, o protagonismo de profissionais enfermeiros como detentores de conhecimentos e capacitados à ministração deste^(17)^.

Atendendo às necessidades da porção acadêmica e geral da população, simultaneamente, Gilbert et al.^(18)^ discorrem acerca da prospecção de conceitos-chave, essenciais à vivência dos grupos de profissionais da saúde, promotores de assistência, e de cidadãos, relacionados à hepatite A. Para tal, fora realizada a estratificação dos grupos, sendo estes interrogados sobre os conceitos ditos de maior importância, adjuntos a um grupo de *experts* na doença. Ficou clarificado o ligeiro despreparo do nível assistencial sobre determinados conceitos, os quais a população demonstrou indispensáveis, havendo, posteriormente, a proposição de uma *checklist* para produção de material educativo^(18)^.

Na observância da diversidade das práticas educacionais, individuais e coletivas, Nyamathi et al.^(20)^ descreveram o impacto de sessões expositivas, ministradas por enfermeiros, sobre hepatite A e outras IST, além de efeitos adversos à vacinação e frequência vacinal em um programa de imunização de pessoas em situação de rua. Ademais às sessões sobre prevenção e promoção de saúde, temáticas comportamentais e psicossociais demonstraram-se influentes na aceitação e completude do esquema vacinal proposto^(20)^, novamente verificando a pertinência do profissional enfermeiro em atividades educativas e imunizadoras.

O gerenciamento individual de casos conduzido por profissionais de enfermagem e associado a práticas educativas, classificado como tecnologia leve-dura, mostrou-se benéfico para promover comportamentos de saúde, como a vacinação e a regularização do esquema vacinal, a testagem sorológica, a adoção de práticas sexuais seguras e a adesão a programas de tratamento para o uso de drogas^(22)^. Neste âmbito, é demonstrada a capacidade educativa da enfermagem, com abrangências individuais ou massivas. Complementarmente ao gerenciamento individual de caso, a formação de *peer coaching*, consideradas duplas de apoio mútuo, demonstrou-se de imensurável valia em um programa de controle de uso crônico de drogas, sobre o qual objetivava-se verificar a propensão à completude vacinal contra hepatite A^(22)^.

A partir das tecnologias identificadas, denota-se a incipiência da produção literária nacional, evidenciando a escassez de abordagens voltadas a populações alvos, como a comunidade LGBTQIAPN+, trabalhadores expostos a riscos e pessoas em situação de rua, como verificado internacionalmente. Propõem-se, assim, iniciativas para a imunização em massa desses grupos vulneráveis, e evidencia-se a necessidade de desenvolvimento de um arcabouço científico e econômico, especialmente no âmbito da Atenção Primária à Saúde, visando à criação de campanhas educativas tanto direcionadas quanto gerais. Para a *práxis* clínica de enfermagem, ações como o rastreamento e triagem sorológica de indivíduos com risco iminente de infecção, a verificação da imunização atual e o tratamento adequado a partir do diagnóstico devem ser parte das medidas essenciais para o controle e prevenção da hepatite A.

Puderam-se perceber possíveis limitações acerca da pesquisa, como a escassez de amostras acerca do tema supracitado, restringindo a análise e comprometendo os resultados; a diversidade de metodologias, dificultando a análise e interpretação dos dados, o que gera uma dependência de materiais complementares e literatura cinzenta, reduzindo o acesso às informações não publicadas, confirmando-se então, a necessidade de investigações futuras a fim de fortalecer e ampliar os resultados.

A insipiência literária relacionada às competências legais e práticas do profissional enfermeiro na abordagem da hepatite A, sobretudo no cenário nacional, incita a prospecção da aplicação e documentação de tecnologias em saúde ligadas à doença. Produções literárias tocantes à temática não somente favorecem a adoção de modelos assistenciais e ações cientificamente embasadas, como também atestam sua legalidade durante o exercício e utilização por profissionais de enfermagem.

O mapeamento das tecnologias em saúde oferece amplo espectro de benefícios: ao grupo de profissionais enfermeiros, a partir da divulgação científica de práticas para adoção e aplicação; à comunidade acadêmica, decorrente da verificação da literatura existente, levantando hipóteses e conclusões acerca do tema; à população geral, pelo aprimoramento das ações assistenciais relacionadas à doença. Dessa forma, o estudo conduzido cumpre as necessidades das populações supracitadas, promovendo a visão concisa das tecnologias verificadas.

## CONCLUSÃO

O presente estudo destaca as intervenções voltadas à prevenção e controle da hepatite A, com ênfase nos diferentes níveis de atenção à saúde. A Atenção Primária se destaca como o principal foco, abordando principalmente a vacinação e a educação em saúde. Já a Atenção Secundária e Terciária concentram-se na triagem clínica e na continuidade do cuidado, incluindo a implementação de lembretes eletrônicos e protocolos sistematizados para testes e monitoramento da vacinação em pacientes institucionalizados. Nesse contexto, é importante ressaltar o papel das tecnologias de saúde utilizadas pela enfermagem, que envolvem tanto as tecnologias leves (como o suporte e a promoção de interações), quanto as leves-duras e duras (como a vacinação e os lembretes eletrônicos).

A concentração dos estudos de origem estadunidense reforça a percepção de insipiência literária, contrastando sistemas de saúde e Atenção Primária à Saúde capazes de abarcar demandas de rastreio, prevenção e tratamento da doença em suas incumbências como o SUS – e a possível negligência e falta de preparo técnico na prevenção e controle da Hepatite A.

No que se refere aos aspectos sociodemográficos, o estudo destaca estratégias direcionadas ao público LGBTQIAPN+, pessoas em situação de rua e trabalhadores expostos a condições de risco ou vulnerabilidade. Por fim, no âmbito cultural, enfatizam-se as campanhas educativas com o objetivo de conscientizar a população sobre práticas sexuais seguras e medidas preventivas. Essa abordagem integrada visa melhorar a cobertura e a eficácia das ações de saúde, promovendo um cuidado mais acessível e eficaz para as populações chaves.

## Data Availability

Os dados encontram-se disponíveis no OSF: https://www.doi.org/10.17605/OSF.IO/MJESK.

# Translation: Tecnologías de enfermería en salud para la prevención y el control de la hepatitis A: una revisión de alcance

**DOI:** 10.1590/1980-220X-REEUSP-2025-0051es

**Authors:** Leonardo Cassimiro Fonseca e Souza, Camila Stela Gomes, Laís Oliveira de Moraes Tavares, João Pedro Vasconcelos Paolinelli, João Marcos Alves Melo, Valéria Conceição de Oliveira, Brener Santos Silva, Gabriela Gonçalves Amaral

**Affiliations:** 1 Universidade do Estado de Minas Gerais, Divinópolis, MG, Brasil.; 2 Universidade Federal de São João del-Rei, Divinópolis, MG, Brasil.; 3 Universidade do Estado de Minas Gerais, Passos, MG, Brasil.; 4 Universidade de São Paulo, São Paulo, SP, Brasil.

**Keywords:** Hepatitis Viral Humana, Hepatitis A, Prevención de Enfermedades, Control de Enfermedades Transmisibles, Enfermería

## Abstract

**Objetivo:**

Mapear la evidencia científica sobre las tecnologías de salud de enfermería en la prevención y control de la Hepatitis A.

**Método:**

Revisión del alcance realizada en 2024 de acuerdo con las recomendaciones del JBI e informada siguiendo los criterios PRISMA-ScR. La búsqueda se realizó (06/10/2024) en bases de datos y literatura gris. Las tecnologías encontradas se clasificaron en: blanda, blanda-dura y dura. Los resultados fueron analizados descriptivamente y sintetizados.

**Resultados:**

Se seleccionaron diez estudios, todos originarios de los EE.UU. Entre las tecnologías se encuentran la vacunación; pruebas serológicas; educación para la salud; recordatorios en los sistemas de información sanitaria; formación de *peer coaching*; gestión de casos; consultas; notificación de enfermedades y lesiones; cribado clínico; y seguimiento y vigilancia. Las tecnologías duras prevalecieron.

**Conclusión:**

La Atención Primaria de Salud se destacó como el foco principal, abordando principalmente la vacunación y la educación para la salud. Atención Secundaria y Terciaria se centraron en el triaje clínico y la continuidad de la atención. La concentración de estudios de origen estadounidense refuerza la percepción de ineptitud literaria y posible negligencia y falta de preparación técnica en la prevención y control de la Hepatitis A.

## INTRODUCCIÓN

La hepatitis A es causada por el virus A, que pertenece a la familia *Picornaviridae* y al género *Hepatovirus*, y es frecuentemente referida como «hepatitis infecciosa»^(1)^. Los síntomas, cuando se presentan, suelen ser inespecíficos, empezando por fatiga, malestar, fiebre y dolor muscular, seguidos de manifestaciones gastrointestinales como náuseas, vómitos, dolor abdominal, estreñimiento o diarrea. Antes de la ictericia puede aparecer orina oscura. Los síntomas suelen aparecer entre 15 y 50 días después de la infección y tienden a durar menos de dos meses^(2)^.

Durante la fase aguda, se produce la replicación viral inicial, acompañada de la eliminación del virus en las heces. La transmisibilidad puede producirse desde dos semanas antes hasta al menos una semana después de la aparición de ictericia u otros síntomas clínicos, además de la elevación de las enzimas hepáticas. El virus puede detectarse en las heces alrededor de una o dos semanas después de la exposición, persistiendo una media de 79 días tras el pico de infección^(3-5)^.

La infección en personas mayores de 50 años tiende a progresar de forma más grave y sintomática, con ictericia en más del 70% de los pacientes. El periodo de incubación es de 28 días de media, con un rango de 15 a 50 días. Aunque no hay informes de hepatitis A que desemboquen en hepatitis crónica o hepatocarcinoma, pueden darse casos de hepatitis fulminante^(4,5)^. En raras ocasiones se producen complicaciones, la más grave de las cuales es la insuficiencia cardiaca fulminante, que ocurre en el 0,5% de los casos, así como manifestaciones extrahepáticas como hemólisis, colestasis acalculosa, derrames pleurales y pericárdicos, artritis reactiva, pancreatitis y manifestaciones neurológicas^(1)^.

La hepatitis A se transmite principalmente por vía oral-fecal, asociada al consumo de alimentos o agua contaminados, y se ve influida por los bajos niveles de saneamiento básico e higiene personal. Otras formas de transmisión incluyen el contacto personal estrecho (como entre personas que viven en la misma casa o niños en guarderías), así como el contacto sexual, especialmente por vía oro-anal o dígito-anal^(4)^. Entre las poblaciones con mayor riesgo de contraer el virus por contacto sexual, los hombres que tienen sexo con hombres (HSH) son un grupo que contribuye al aumento de la incidencia, incluidos los hombres homosexuales, bisexuales, no binarios y heterosexuales que tienen sexo con otros hombres^(6).^

A nivel mundial, se ha producido un aumento significativo de la incidencia de hepatitis A aguda de transmisión sexual, especialmente entre los HSH. En la Unión Europea, se documentaron 19 947 casos entre 2016 y 2018, lo que representa un aumento de cuatro veces en comparación con el período de tres años anterior (2012 a 2015)^(7)^. En Brasil, entre 2000 y 2021, se notificaron 718 651 casos confirmados de hepatitis viral, de los cuales 168 175 (23,4%) fueron de hepatitis A. A pesar de la reducción global de las notificaciones, que alcanzó el 95,6% entre 2011 y 2021^(8)^, este descenso se debe en gran medida a la amplia cobertura de vacunación ofrecida gratuitamente a los niños de hasta 4 años, 11 meses y 29 días^(9)^. Sin embargo, en regiones específicas, como el estado de Río de Janeiro, se observó un aumento significativo en 2018, con 9 casos por 100 000 habitantes, en contraste con los 0,3 casos por 100 000 habitantes del año anterior. Este aumento fue especialmente notable entre la población de HSH^(10)^.

Además, la naturaleza de la transmisión de la hepatitis A está influenciada por los desastres naturales que afectan a la distribución de alimentos y agua. La recurrencia de inundaciones, como las ocurridas en Rio Grande do Sul en mayo de 2024, favorece la diseminación de la enfermedad, destacando factores sociodemográficos que contribuyen a la propagación^(11)^. Esto enfatiza la urgencia de implementar medidas efectivas de control y prevención.

Los cuidados de enfermería son fundamentales en todas las acciones de control y prevención de la infección, abarcando diversas áreas. Esto incluye el sistema de vigilancia de las hepatitis víricas, que monitoriza las tendencias de la enfermedad; la planificación de estrategias de prevención y control; el cribado clínico de los posibles donantes de sangre y hemoderivados en los bancos de sangre; la administración de vacunas y las campañas de inmunización; la atención a las mujeres embarazadas para prevenir la transmisión vertical; la detección de casos de hepatitis víricas; la atención terciaria a las personas con formas agudas y crónicas de la infección; y la producción de conocimientos que permitan la aplicación segura de prácticas basadas en la evidencia^(12)^.

Es importante destacar que la hepatitis A está menos cubierta en comparación con otras infecciones como la hepatitis B, la sífilis y el VIH, entre otras infecciones de transmisión sexual (ITS). Esto se traduce en una importante falta de conocimientos técnicos y prácticos y de sistematización de la atención a los pacientes afectados por esta infección. Además, existe una falta de liderazgo y participación por parte de las enfermeras en el abordaje de la hepatitis A, especialmente teniendo en cuenta su transmisión sexual y las especificidades de morbilidad y mortalidad. Por lo tanto, es necesario que los profesionales de enfermería se impliquen en la prevención de la hepatitis A y en la promoción de la salud.

Por lo tanto, este estudio tiene como objetivo mapear la evidencia científica sobre las tecnologías en salud de enfermería en la prevención y control de la hepatitis A.

## MÉTODO

### Diseño del Estudio

Se trata de una revisión de alcance realizada de acuerdo con las recomendaciones del *Joanna Briggs Institute* (JBI) – *Manual for Evidence Synthesis for Scoping review* ^(13)^; y reportada siguiendo los criterios de la *extensión de los Preferred Reporting Items for Systematic reviews and Meta-Analyses for Scoping Reviews* (PRISMA-ScR)^(14)^. El protocolo de revisión está registrado en el *Open Science Framework* (OSF) *Registries* (https://doi.org/10.17605/OSF.IO/MJESK).

### Fuentes de Datos y Estrategia de Búsqueda

En mayo de 2024 se realizó una búsqueda preliminar en las bases de datos OSF y PROSPERO del *National Institute for Health and Care Research* para mapear y verificar estudios similares en curso o finalizados, evaluando el alcance y la metodología utilizada, sin encontrar registros relevantes.

Para definir la pregunta guía se utilizó la nemotecnia Población-Concepto-Contexto (PCC). Se consideró población (P) a los profesionales de enfermería; concepto (C) a las tecnologías utilizadas en la prevención y control de la hepatitis A; y contexto (C) a todos los niveles asistenciales. Las tecnologías fueron consideradas como las diferentes habilidades técnico-prácticas utilizadas por los profesionales de enfermería durante la atención, ya sea que se refieran a la atención clínica, a la educación, a la vigilancia epidemiológica, a la prevención y promoción de la salud, o incluso a las tecnologías físicas y materiales. Así, la pregunta orientadora fue: «¿Cuáles son las tecnologías sanitarias utilizadas por los profesionales de enfermería en la prevención y control de la hepatitis A?».

Las búsquedas fueron realizadas en el Portal de la Biblioteca Virtual en Salud (BVS), incluyendo *la Biblioteca Nacional* de Medicina (NLM – MEDLINE)/*Biblioteca Nacional de Medicina e Institutos Nacionales de Salud* (PubMed); *Ciencias de la Salud de América Latina y el Caribe* (LILACS); Índice Bibliográfico Español en Ciencias de la Salud (IBECS); y Base de Datos de Enfermería (BDEnf). Además, se consultaron las bases de datos PubMed/Medline; EMBASE; Scopus; *Web of Science* (WOS); CINAHL *Database*; y *Cochrane Library*. Para la literatura gris, se consultó el Catálogo de *Tesis* y Disertaciones de la Coordinación para la Mejora del Personal de Educación Superior (CAPES); *Theses Canada;* y *World Cat Dissertations* and *Theses*. Por último, se comprobaron las referencias de los estudios analizados en busca de bibliografía complementaria.

La búsqueda se dividió en etapas: a) una búsqueda preliminar para comprobar la existencia de estudios similares en curso, junto con la identificación de términos y palabras clave de búsqueda; b) la recopilación de términos y palabras clave, seguida de la formulación de estrategias de búsqueda y su adaptación a las especificidades de las bases de datos consultadas; c) la comprobación de los resultados y la selección de los registros utilizados. Las estrategias de búsqueda, adaptadas a cada base de datos, se publican como material complementario, disponible en https://doi.org/10.17605/OSF.IO/MJESK.

Se decidió incluir artículos originales con abordaje cuantitativo, cualitativo o mixto; disponibles en su totalidad; sin restricciones de tiempo; en portugués, inglés y español; que abordaran tecnologías utilizadas por los profesionales de enfermería en la prevención y control de la hepatitis A. También se incluyeron informes de experiencias. Se excluyeron revisiones bibliográficas, protocolos de estudio, cartas, editoriales y carteles. Además, se descartaron los estudios de naturaleza puramente epidemiológica, los estudios no relacionados con la población humana o que trataran de productos alimenticios.

### Recleccción y Extracción de Datos

Tras consultar las bases de datos el 10/06/2024, los registros obtenidos se importaron al *programa informático Rayyan*, que ayuda a la selección, empezando por la eliminación de duplicados. Dos revisores independientes realizaron un cribado por título y resumen. A partir de esta selección, los textos fueron analizados en su totalidad para verificar su pertinencia y el cumplimiento de los criterios de elegibilidad, por dos revisores independientes, siguiendo el método de revisión doble ciego. En caso de impasse o dudas, se consultó a un tercer revisor. De los registros excluidos por no estar disponibles en su totalidad, se intentó contactar por *correo electrónico* con los autores de los estudios para solicitar el trabajo, sin obtener respuesta. Se utilizó el diagrama de flujo PRISMA-ScR^(14)^ para informar de los resultados de la búsqueda y registrar los motivos de los artículos excluidos.

Se utilizó una herramienta de recogida de datos desarrollada por los autores, que fue evaluada mediante una prueba piloto con datos extraídos de cinco artículos para su calibración. Las variables recogidas incluyeron: título, autor(es), país, año de publicación, objetivo(s), diseño del estudio, participantes, ámbito del estudio, nivel de atención sanitaria, tecnologías para la prevención y control de la hepatitis A, clasificación de las tecnologías, así como conclusiones y recomendaciones. De acuerdo con las recomendaciones del JBI^(15)^, no se analizó individualmente el riesgo de sesgo de los estudios seleccionados.

La clasificación de las tecnologías se basó en el marco teórico descrito por Merhy^(16)^, y se dividieron en tecnologías blandas, que se refieren a las relaciones humanas, como la producción de vínculos y relaciones, la autonomía, la acogida, la gestión de los procesos de trabajo; blandas-duras, como en el caso de los conocimientos bien estructurados que operan en el trabajo en salud, como la clínica médica, el psicoanálisis, la epidemiología, entre otros; y duras, como en el caso de los equipos tecnológicos, las máquinas, las normas y las estructuras organizativas.

### Síntesis de Los Resultados

A partir de los datos extraídos, se elaboró un resumen de las pruebas, organizado en tablas, junto con la interpretación y síntesis general de los resultados, destacando las lagunas de conocimiento. También se analizaron las aportaciones de las tecnologías disponibles, así como las sugerencias para las políticas públicas y la evolución de la práctica clínica de enfermería.

### Aspectos Éticos

Al tratarse de una revisión de alcance, no fue necesario someter el estudio a un Comité de Ética de la Investigación.

## RESULTADOS

Las búsquedas en las bases de datos arrojaron 3.453 registros. Después de excluir 815 duplicados, quedaron 2.638 archivos posiblemente elegibles para la revisión. Se leyeron los títulos y resúmenes para comprobar los criterios de elegibilidad, lo que resultó en la exclusión de 2.608 publicaciones y dejó 30 estudios para ser leídos en su totalidad. Finalmente, se seleccionaron 10 estudios. Los detalles de la búsqueda, identificación y selección de los estudios se describen en la Figura 1.

**Figura 1:** Flujograma de identificación, selección, elegibilidad e inclusión de estudios, basado en las recomendaciones PRISMA Divinópolis, MG, Brasil, 2024.

Los estudios fueron publicados entre 2004 y 2022, con mayor ocurrencia en 2015 (n = 2; 20%) y 2020 (n = 2; 20%). En cuanto a la nacionalidad, todas las publicaciones tuvieron lugar en los Estados Unidos, en particular en los estados de Pensilvania (n = 3; 30%) y California (n = 2; 20%). En cuanto al diseño de los estudios, cuatro (40%) fueron informes de experiencia; dos (20%) estudios transversales; un (10%) estudio cuasi-experimental; un (10%) ensayo clínico aleatorizado; un (10%) estudio descriptivo cualitativo; y un (10%) estudio de cohortes retrospectivo. En cuanto a los niveles de atención sanitaria, destacaron la Atención Primaria de Salud (APS) (n = 5; 50%) y la Atención Secundaria de Salud (n = 2; 20%). Además, hubo una intersección entre APS y Secundaria (n = 1; 10%) y APS y Terciaria (n = 1; 10%). Sólo un estudio (10%) se realizó utilizando como telón de fondo un medio de comunicación popular: la televisión (Cuadro 1).

**Cuadro 1:** Tecnologías en salud en la prevención y control de la Hepatitis A – Divinópolis, MG, Brasil, 2024.

Título autor(es) año ciudad/país de origen		Objetivo(s)	Diseño	Participantes	Escenario Nivel de asistencia de salud	Tecnologías para prevención y control de Hepatitis A	Clasificación de la tecnología	Contribuciones para la práctica
**E1**	Nurses’ use of the media to provide public health information during a hepatitis A outbreak Davidson y George^(17)^ Moon Township, Pennsylvania, EUA 2004	Relatar la experiencia realizada	Relato de experiencia	Telespectadores	Medios de comunicación: televisión	- Educación en salud llevada a cabo en una cadena de televisión por tres enfermeras sobre el HEP A: transmisión, periodo de incubación, lavado de manos, signos y síntomas, actuación en caso de síntomas, diferencias entre vacuna e inmunoglobulina.	Liviana-duras	- La oportunidad no sólo puso en valor a la universidad y a los profesionales implicados, sino que también demostró el carácter científico-informativo de las enfermeras en temas de salud pública
**E2**	Viral hepatitis prevention education: What do people and providers need to know? Gilbert et al.^(18)^ Atlanta, Georgia, EUA. 2005	- Elaborar una lista consensuada de conceptos clave para los materiales educativos sobre las hepatitis A, B y C destinados a pacientes y profesionales sanitarios; - Verificar qué debe saber la población general sobre la hepatitis vírica; - Verificar lo que los profesionales sanitarios deben saber sobre la hepatitis vírica; - Elaborar una lista de conceptos esenciales para la población general y los profesionales en salud; - Comparar las recomendaciones para la población general y los profesionales en salud.	Estudio cualitativo realizado con tres poblaciones: *expertos* en hepatitis víricas, profesionales sanitarios y población general.	11 *expertos* en hepatitis víricas; 10 profesionales sanitarios, de los cuales sólo una enfermera; y 158 personas ajenas a los grupos anteriores, de edades comprendidas entre los 18 y los 49 años, en su mayoría mujeres.	Atención primaria de salud Atención terciaria de salud	- Elaboración de preguntas sobre la HEP vírica, desde la perspectiva de *expertos*, profesionales sanitarios y población general, con el objetivo de verificar conceptos clave esenciales en los conocimientos de cada grupo observado; - Formulación de una lista de comprobación para orientar la educación en salud sobre la HEP vírica.	Leve-duras	- El estudio aportó información acerca de que los conceptos clave para elaborar material informativo deben proceder de diversas fuentes, incluido el público al que va dirigido; - Para futuros estudios, se sugiere que se incluyan diversos temas relacionados con la comunicación en salud, como el alcance y la fiabilidad de las fuentes, y que se tengan en cuenta criterios sociodemográficos como la etnia/raza y la orientación sexual a la hora de llevar a cabo la educación en salud.
**E3**	Hepatitis in primary care: what NPs can do to save lives Stonsifer et al.^(19)^ Pennsylvania, EUA 2006	Informe sobre la práctica asistencial realizada	Relato de experiencia	3 expertos: una enfermera, un profesor de medicina y un investigador en epidemiología	Atención primaria de salud	- Pruebas en pacientes durante la anamnesis, especialmente en aquellos con anomalías de las enzimas hepáticas; antecedentes de consumo de drogas inyectables; transfusiones anteriores a 1992; personas privadas de libertad; individuos procedentes de África, Asia o Europa del Este; antecedentes de contactos sexuales de riesgo o con hombres que practican sexo con hombres; - Vacunación contra el HEP A y B para personas con hepatopatía crónica; mayor riesgo sexual; consumo de drogas y drogas inyectables; viajeros o trabajadores de países con riesgo de contracción; trabajadores sanitarios y de seguridad pública; trabajadores de guarderías y trabajadores de alcantarillado.	Duras	- Necesidad de vacunación contra la hepatitis A y B en poblaciones de riesgo. - Colaboración enfermera en el diagnóstico inicial y atención primaria, educación en salud en relación con las hepatitis A y B y exploración del paciente durante la recogida de información sobre su historia clínica.
**E4**	Effects of a nurse-managed program on hepatitis A and B vaccine completion among homeless adults Nyamathi et al.^(20)^ Los Angeles, California, EUA 2009	- Pruebas en pacientes durante la anamnesis, especialmente en aquellos con anomalías de las enzimas hepáticas; antecedentes de consumo de drogas inyectables; transfusiones anteriores a 1992; personas privadas de libertad; individuos procedentes de África, Asia o Europa del Este; antecedentes de contactos sexuales de riesgo o con hombres que practican sexo con hombres; - Vacunación contra el HEP A y B para personas con hepatopatía crónica; mayor riesgo sexual; consumo de drogas y drogas inyectables; viajeros o trabajadores de países con riesgo de contracción; trabajadores sanitarios y de seguridad pública; trabajadores de guarderías y trabajadores de alcantarillado.	Estudio prospectivo, cuasiexperimental y aleatorio realizado con tres grupos de adultos viviendo en la calle	865 adultos sin hogar, de 18 a 65 años, capaces y dispuestos a recibir la inmunización contra la PPE A y B, en un programa de inmunización inicial y que regresan al cabo de seis meses.	Atención primaria de salud	- Educación en salud sobre el HEP A; B, C y el VIH: vías de transmisión; diagnóstico; prevención; nociones sobre la vacuna contra el HEP A y B; calendario de vacunación; posibles reacciones y efectos secundarios; así como su importancia; - Educación en salud sobre temas como la autoestima, la comunicación, el comportamiento, el consumo de drogas, el comportamiento sexual y las influencias para completar el calendario de vacunación contra las HEP A y B; - Seguimiento; - Pruebas de HEP A, B, C y VIH. - Vacunación contra el HEP A y B.	Leve-duras, duras	El programa mostró una mejora significativa en las vacunaciones durante un periodo de seis meses (0, 180 días) contra el HEP A y B en comparación con el control estándar La estrategia de enfermería fue clave para este aumento
**E5**	Integrating Health and Prevention Services in Syringe Access Programs: A Strategy to Address Unmet Needs in a High-Risk Population Burr et al.^(21)^ New Jersey, EUA 2014	Describir la aplicación de un proyecto dirigido por enfermeras para la promoción de la salud y la prevención de enfermedades, diseñado inicialmente para acercarse a las mujeres en programas de intercambio de jeringuillas para la atención prenatal y la atención del VIH.	Estudio transversal	3488 consumidores de drogas por vía parenteral, el 38% de los cuales eran mujeres, que visitaron las instalaciones del programa de intercambio de agujas.	Atención primaria de salud	- Programa dirigido por enfermeros para la prevención y el control de las ITS, así como la promoción de la salud materno-fetal para portadores del VIH y personas con riesgo de contraerlo; - Pruebas de ITS; - Vacunación contra el HEP A y B (*Twinrix*).	Leve-duras, duras	- El estudio demostró que la inclusión de servicios de prevención y promoción de la salud dirigidos por enfermeras en los servicios de intercambio de agujas llega a un gran número de consumidores de drogas inyectables y mujeres embarazadas; Además de la promoción de la salud mediante el intercambio de agujas, la inclusión de otros servicios, como las pruebas y el tratamiento del VIH y la hepatitis, ha ampliado el alcance, especialmente a poblaciones marginadas como los consumidores de drogas inyectables.
**E6**	Nursing case management, peer coaching,and Hepatitis A and B vaccine completion among homeless men recently released on parole: randomized clinical trial Nyamathi et al.^(22)^ Los Angeles, California, EUA 2015	- Identificar qué intervención (*asesoramiento intensivo entre iguales* e intervención de casos dirigida por enfermeras; *asesoramiento intensivo entre iguales*; y atención habitual) es más asertiva a la hora de completar el calendario de vacunación HEP A y B; - Identificar los factores predictivos de la finalización de la vacunación HEP A y B.	Ensayo clínico randomizado	345 personas en libertad condicional incluidas en residencias de tratamiento de drogodependencias	Atención secundaria de salud	- *Coaching entre iguales*; - Gestión de casos, en sesiones con una enfermera, durante 45 minutos/semana, durante 8 semanas, basada en la finalización de la vacunación, la adherencia al programa de rehabilitación de drogodependientes, la promoción de la salud y la reducción del riesgo sexual y del abuso de sustancias; - Vacunación contra el HEP A y B (*Twinrix*) siguiendo el calendario de 0, 7 y 21-30 días.	Leves, leves-duras, duras	- Cumplimiento del programa de vacunación contra el HEP A y B. - Evaluación de la eficacia de tres intervenciones llevadas a cabo por enfermeras, con similitudes entre las modalidades de atención propuestas; - Desarrollo de varios niveles de programas dirigidos por pares y por enfermeras para mejorar la aceptación de la vacuna HEP A y B. - Inclusión de enfermeras en residencias de tratamiento de drogodependencias
**E7**	Viral Hepatitis: New U.S. Screening Recommendations, Assessment Tools, and Treatments Dan et al.^(23)^ Washington, EUA 2015	- Analizar la epidemiología y el diagnóstico de la HEP A, la SHEP B y la HEP C; - Discutir la historia natural de la HEP B y la HEP C crónicas; - Describir el plan de acción del Departamento de Salud y Servicios Humanos de EE.UU., centrado en el papel de las enfermeras en la prevención, el tratamiento y, en el caso del VHC, la curación	Relato de experiencia	- Personas infectadas por el VIH; - Cualquier persona que haya consumido drogas inyectables (incluso una vez); - Trabajadores sanitarios, personal de urgencias médicas y trabajadores de seguridad pública tras lesiones por pinchazo de aguja o exposición de mucosas a sangre contaminada con HEP C; - Niños nacidos de madres que han dado positivo en la prueba del HEP C;	Atención primaria de salud	- Cribado serológico; - Proceso de cribado en dos fases (prueba de anticuerpos y prueba de ácidos nucleicos).	Duras	- Fomento de la educación en salud; - Revisión de los planes de acción contra las hepatitis víricas; - Destacar la importancia del papel de la enfermera en el control de la infección, el desarrollo y las prácticas relacionadas con los planes de acción antes mencionados.
**E8**	Hepatitis A takes hold in the community Heavey^(24)^ Brockport, New York, EUA. 2020	Describir las infecciones por virus HEP A en personas de riesgo, como los sin techo, en Estados Unidos.	Relato de experiencia	Población general	Atención primaria de salud	- Educación en salud sobre lavado de manos, higiene de superficies con cloro, consumo adecuado de alimentos y agua; - Vacunación contra HEP A (*Havrix y Vaqta*) y contra HEP A y B (*Twinrix*); - Notificación a los sistemas de vigilancia epidemiológica y de salud pública; - Consulta a los viajeros; - Pruebas para los viajeros.	Leve-duras, duras	Las enfermeras tienen un papel que desempeñar en la educación y los cuidados, especialmente con la inmunización, así como en la prevención y el diagnóstico de la HEP A.
**E9**	Emergency Department-based Hepatitis A Vaccination Program in Response to an Outbreak Kaigh et al.^(25)^ Philadelphia, EUA 2020	Evaluación de la incidencia de nuevos casos de HEP A tras la aplicación de un programa de vacunación guiado por *el consejo de buenas prácticas* (BPA).	Estudio descriptivo transversal retrospectivo	Datos de 5009 personas que acudieron a un servicio de urgencias, percibidas como población de riesgo (personas viviendo en la calle, consumidores de drogas, hombres que tienen relaciones sexuales con hombres, personas recientemente encarceladas).	Atención primaria de salud Atención secundaria de salud	- Vacunación contra el HEP A; - Uso de BPA, una herramienta que activa un recordatorio, basado en el historial y la historia clínica de cada paciente, con el fin de comprobar el estado de vacunación y la elegibilidad.	Duras	El uso de las tecnologías empleadas, así como la asociación entre la sanidad pública y los servicios de urgencias, contribuyeron a la inmunización de 669 personas, y también se produjo una reducción de la incidencia de HEP A.
**E10**	An electronic medical record-based intervention to improve hepatitis A vaccination rates in the emergency department during a regional outbreak Bukhsh et al.^(26)^ Michigan, EUA 2022	Determinar el éxito de la implantación de la BPA para aumentar la tasa de vacunación frente al HEP A en el servicio de urgencias de un hospital, así como evaluar cuantitativamente el uso y las barreras de la implantación.	Estudio de cohortes retrospectivo y *análisis de encuestas*.	11.016 pacientes ingresados en un servicio de urgencias, examinados y verificados como individuos en riesgo de HEP A (personas sin hogar, consumidores de drogas, presos, pacientes con enfermedades hepáticas y HSH).	Atención secundaria de salud	- La BPA insta a la vacunación de las personas con riesgo de contraer el HEP A; - Vacunación contra la HEP A; - Pruebas serológicas.	Duras	La herramienta utilizada es eficaz para los recordatorios de vacunación contra el HEP A, pero necesita la opinión de los usuarios (médicos, cuidadores y enfermeros) para futuras mejoras. Durante el periodo de uso de la herramienta se produjo un aumento masivo del número de solicitudes de vacunas.

Nota: EUA.: Estados Unidos de América; HEP: hepatitis; VIH: virus de la inmunodeficiencia humana; ITS: infecciones de transmisión sexual; BPA: Best Practice Advisory – *(recomendación de buenas prácticas)*.

En cuanto a las tecnologías analizadas, se formó un grupo diverso, teniendo en cuenta las diferentes áreas en las que trabajan los profesionales de enfermería, a saber: vacunación^(19–21,24–26)^; pruebas serológicas^(19–21,23,24,26)^; educación sanitaria^(17,18,20,21,24)^; uso de recordatorios en sistemas de información sanitaria^(25,26)^; peer coaching (*coaching entre iguales*) en residencias de tratamiento de consumo crónico de drogas^(22)^; gestión de casos^(22)^; consultas^(24)^; notificación de enfermedades y dolencias^(24)^; cribado clínico^(19)^; y seguimiento y cribado^(20)^ (Figura 2).

**Figura 2:** Tecnologías en salud en la prevención y control de la hepatitis A Divinópolis, MG, Brasil, 2024.

En cuanto a la clasificación de las tecnologías, las tecnologías duras incluyeron la vacunación; las pruebas; el cribado clínico; y el uso del *Best Practice Advisory* (BPA), un recordatorio electrónico en los sistemas de información sanitaria. Las tecnologías blandas-duras encontradas permeaban la educación en salud; la producción de una *lista de verificación* para orientar el proceso de educación en salud; el monitoreo y tamizaje de la población general y de riesgo; la gestión de casos realizada por enfermeros; la notificación a los sistemas de vigilancia epidemiológica; y las consultas para grupos propensos a la contracción. Como resultado, las tecnologías blandas, por su caracterización social y relacional, denotaron la formación de *coaching entre pares*, descritos como pares de apoyo mutuo (Cuadro 1).

## DISCUSIÓN

Fueron abordadas las tecnologías disponibles en la literatura, que los profesionales de enfermería tienen competencia legal y práctica para utilizar en la prevención y control de la hepatitis A, en todas las áreas de atención a la salud. Se observó heterogeneidad en las tecnologías utilizadas, así como en su uso en los diferentes niveles de atención a la salud, predominando el uso de tecnologías clasificadas como duras.

Entre las tecnologías duras destacó la vacunación, con mayor recurrencia y uso. Se han descrito dos tipos de inmunizantes: la vacuna combinada adsorbida contra la hepatitis A y B (*Twinrix* ^©^), y las vacunas adsorbidas inactivadas contra la hepatitis A (*Vaqta* ^©^ y *Havrix* ^©^), recomendadas y puestas a disposición por el Advisory Board on Immunisation Practices de los Centers for Disease Control and Prevention de los EUA^(24,27)^. Según Soares et al.^(28)^, las enfermeras tienen conocimientos sobre almacenamiento, distribución, administración, efectos adversos y medidas de eliminación de residuos, lo que las convierte en actores clave e indispensables durante todo el proceso de vacunación.

La inmunización con la vacuna *Twinrix* ^©^ resulta ventajosa en comparación con los demás inmunizantes utilizados, dado que se mantiene el calendario estándar de seis meses, en el que se administra la dosis inicial y dos refuerzos a los 30 y 180 días de la primera dosis. También destaca la inmunización contra la hepatitis A y B con un solo inmunizante^(20)^. Además, Nyamathi et al.^(22)^ describen la posibilidad de acortar el calendario de vacunación, siempre con tres administraciones del inmunizante, con refuerzos después de 7 y entre 21 y 30 días después de la dosis inicial. *Havrix* ^©^ y *Vaqta* ^©^, vacunas inactivadas, son equivalentes entre sí y se recomiendan en función del grupo de edad, observando también un calendario específico para cada edad^(24,27)^.

En cuanto a las recomendaciones de vacunas, en el contexto analizado destaca la inclusión de grupos diana, además de las indicaciones de vacunación masiva para grupos de edad específicos. Estas poblaciones incluyen personas viviendo en la calle; individuos con restricción de libertad; población LGBTQIAPN+, especialmente HSH y mujeres transexuales; usuarios de drogas inyectables; trabajadoras sexuales; viajeros a áreas de riesgo; trabajadores de alcantarillado; y trabajadores de guarderías^(19,22,24,29)^. Cabe destacar que, en Brasil, la vacuna contra la hepatitis A está disponible a través del Sistema Único de Salud (SUS), enfocándose en la población objetivo de niños entre 15 meses y cinco años de edad, en dosis única^(30)^; también se inmuniza a grupos específicos, como inmunodeprimidos, pacientes crónicos con hepatitis B, personas que viven con VIH o SIDA y otras condiciones específicas, a través de la derivación a Centros de Referencia de Inmunobiológicos Especiales^(31,32)^.

También se ha hecho hincapié en la influencia de un fenómeno conocido como vacilación vacunal, o conjunto de variables que provocan retrasos y rechazo de la vacuna, en la vacunación contra la hepatitis A, dadas las bajas tasas de cobertura y la epidemia de la enfermedad. Magyar et al.^(33)^ describieron la indecisión vacunal de los padres en niños de entre cero y dos años en Austria, observando un mayor rechazo a la vacuna de la hepatitis A en comparación con otras vacunas. El estudio destacaba factores económicos, en particular ante la necesidad de pagar la vacuna, así como razones logísticas e ideológicas, que tienden a validar la aparición de infecciones por el virus de la hepatitis A.

En esta perspectiva, aún en referencia a las tecnologías duras, se ha verificado la relevancia de las pruebas serológicas, o tamizaje serológico, para hepatitis A y otras ITS, con foco en poblaciones clave^(19-21,23,24,26)^. Aunque existe una importante falta de bibliografía sobre el cribado serológico de la hepatitis A, Bukhsh et al.^(26)^ describen el proceso de pruebas obligatorias en un servicio de urgencias para personas con una mayor susceptibilidad a la infección por el virus, además de proponer la creación de una herramienta electrónica para los recordatorios de vacunación.

Las herramientas electrónicas, como las descritas por Kaigh et al.^(25)^ y Bukhsh et al.^(26)^, actúan mediante recordatorios en los sistemas de información sanitaria, a partir de *avisos*, o mecanismos automáticos resultantes de una alimentación inicial del sistema, verificando la indicación de vacunación. El GAP cruza los datos serológicos con las variables sociodemográficas, articulando la pertinencia de administrar el inmunizante si el individuo tiene indicación, además de proporcionar información sobre las dosis de refuerzo, en caso necesario^(25,26)^.

Además, el estudio realizado por Ford et al.^(34)^ demuestra el uso de las BPA para el cribado serológico, verificando la ocurrencia de individuos infectados por el virus de la hepatitis C, así como la carga viral, con el objetivo de realizar pruebas asertivas a los pacientes en un servicio de urgencias. La asociación, por lo tanto, entre el uso del BPA para el cribado de poblaciones en riesgo de contraer el virus de la hepatitis A y la vacunación basada en el estado serológico, favorece el control de la transmisión, la inmunización inicial y la verificación de la completitud de la vacuna, integrando una herramienta favorable para la gestión de la enfermedad en todos los ámbitos de la salud.

Como el diagnóstico de las hepatitis virales se realiza a partir de pruebas, los individuos incluidos en grupos de mayor riesgo o que presentan síntomas sugestivos necesitan un cribado serológico. La comprobación de la presencia de inmunoglobulinas específicas para la hepatitis A no sólo permite un diagnóstico preciso (IgM), sino también la inmunización adquirida (IgG), ya sea por vacunación o por infección previa, lo que, no obstante, respalda la indicación de la vacunación^(24)^.

De forma semejante a la vacunación y a las pruebas, el proceso de educación para la salud caracterizado como una tecnología blanda-dura, debido a la agregación de conocimiento y ejecución es parte integrante de la rutina de los profesionales de enfermería, constituyendo una importante herramienta de promoción y prevención de la salud, a través de la orientación, aclaración de dudas y adecuación de estilos y hábitos de vida al estado de salud^(35)^.

Esta tecnología es percibida como de influencia masiva en los medios de transmisión y control preventivo de la hepatitis A. Se describen diferentes formas y escenarios de promoción educativa, abarcando los diferentes grupos propensos a la patología. Se evidenció el uso de los medios de comunicación social de la época la televisión- como medio de comunicación sobre los métodos de infección, la inmunización, los síntomas, la conducta y los tratamientos existentes para la hepatitis A. El uso de medios de comunicación de masas, como la red de televisión, promoviendo el tema de forma popular y dirigida, demostró ser pertinente no sólo para aclarar el virus y sus posibles repercusiones, sino también el papel de las enfermeras profesionales como poseedoras de conocimientos y capacitadas para administrarlos^(17)^.

Atendiendo a las necesidades del mundo académico y de la población en general al mismo tiempo, Gilbert et al.^(18)^ discuten la prospección de conceptos clave esenciales para la experiencia de grupos de profesionales de la salud, promotores de cuidados y ciudadanos, relacionados con la hepatitis A. Para ello, estratificaron los grupos y preguntaron por los conceptos más importantes, junto con un grupo de *expertos* en la enfermedad. Quedó claro que el nivel asistencial estaba poco preparado para determinados conceptos, que la población demostró que eran indispensables, y posteriormente se propuso una *lista de comprobación* para la elaboración de material educativo^(18)^.

Observando la diversidad de las prácticas educativas individuales y colectivas, Nyamathi et al.^(20)^ describieron el impacto de las sesiones expositivas impartidas por enfermeras sobre la hepatitis A y otras ITS, así como sobre los efectos adversos de la vacunación y la frecuencia de vacunación en un programa de inmunización para personas que vivían en la calle. Además de las sesiones sobre prevención y promoción de la salud, los temas conductuales y psicosociales demostraron ser influyentes en la aceptación y cumplimiento del calendario de vacunación propuesto^(20)^, lo que verifica una vez más la relevancia de los profesionales de enfermería en las actividades educativas y de inmunización.

La gestión individual de casos llevada a cabo por profesionales de enfermería y asociada a prácticas educativas, clasificadas como tecnología blanda-dura, ha demostrado ser beneficiosa para promover comportamientos de salud como la vacunación y la regularización del calendario de vacunación, las pruebas serológicas, la adopción de prácticas sexuales seguras y la adherencia a los programas de tratamiento farmacológico^(22)^. En este contexto, queda demostrada la capacidad educativa de la enfermería, con una cobertura individual o masiva. Además de la gestión individual de casos, la formación de *coaching entre pares*, considerados pares de apoyo mutuo, demostró tener un valor inconmensurable en un programa de control del uso crónico de drogas, que tenía como objetivo verificar la propensión a completar la vacunación contra la hepatitis A^(22)^.

A partir de las tecnologías identificadas, existe una incipiente producción literaria nacional, destacándose la escasez de abordajes dirigidos a poblaciones objetivo, como la comunidad LGBTQIAPN+, trabajadores expuestos a riesgos y personas viviendo en la calle, como se observa internacionalmente. Por lo tanto, se proponen iniciativas para la inmunización masiva de estos grupos vulnerables, y es necesario desarrollar un marco científico y económico, especialmente en el ámbito de la Atención Primaria de Salud, con el fin de crear campañas educativas tanto específicas como generales. Para la *práctica* clínica de enfermería, acciones como el cribado y el triaje serológico de los individuos con riesgo inminente de infección, la verificación de la inmunización actual y el tratamiento adecuado tras el diagnóstico deberían formar parte de las medidas esenciales para el control y la prevención de la hepatitis A.

Se pudieron observar posibles limitaciones de la investigación, como la escasez de muestras sobre el tema mencionado, restringiendo el análisis y comprometiendo los resultados; la diversidad de metodologías, dificultando el análisis e interpretación de los datos, lo que genera una dependencia de materiales complementarios y literatura gris, reduciendo el acceso a informaciones inéditas, confirmando así la necesidad de futuras investigaciones para fortalecer y ampliar los resultados.

La escasez de literatura sobre las competencias legales y prácticas de los profesionales de enfermería en el abordaje de la hepatitis A, especialmente en el escenario nacional, motiva la búsqueda de la aplicación y documentación de tecnologías en salud relacionadas a la enfermedad. La literatura sobre el tema no sólo favorece la adopción de modelos y acciones asistenciales con base científica, sino que también atestigua su legalidad durante la práctica y el uso por parte de los profesionales de enfermería.

El mapeo de tecnologías de salud ofrece una amplia gama de beneficios: al colectivo de profesionales de enfermería, a través de la divulgación científica de prácticas para adopción y aplicación; a la comunidad académica, a través de la verificación de la literatura existente, planteando hipótesis y conclusiones sobre el tema; y a la población en general, a través de la mejora de las acciones de cuidado relacionadas con la enfermedad. De esta forma, el estudio realizado atiende a las necesidades de las poblaciones mencionadas, promoviendo una visión concisa de las tecnologías verificadas.

## CONCLUSIÓN

Este estudio destaca las intervenciones dirigidas a la prevención y control de la hepatitis A, con énfasis en los diferentes niveles de atención a la salud. La Atención Primaria se destaca como el foco principal, abordando principalmente la vacunación y la educación en salud. La atención secundaria y terciaria se centran en el cribado clínico y la continuidad asistencial, incluyendo la implantación de recordatorios electrónicos y protocolos sistematizados para la realización de pruebas y el seguimiento de la vacunación en pacientes institucionalizados. En este contexto, es importante destacar el papel de las tecnologías sanitarias utilizadas por la enfermería, que implican tanto tecnologías blandas (como el apoyo y la promoción de interacciones) como tecnologías duras y blandas (como la vacunación y los recordatorios electrónicos).

La concentración de estudios de los Estados Unidos refuerza la percepción de falta de literatura, contrastando sistemas de salud y Atención Primaria de Salud capaces de atender las demandas de cribado, prevención y tratamiento de la enfermedad dentro de sus competencias – como el SUS – y la posible negligencia y falta de preparación técnica en la prevención y control de la Hepatitis A.

En cuanto a los aspectos sociodemográficos, el estudio destaca las estrategias dirigidas al público LGBTQIAPN+, a las personas viviendo en la calle y a los trabajadores expuestos a condiciones de riesgo o vulnerabilidad. Finalmente, en el ámbito cultural, se enfatizan las campañas educativas con el objetivo de concientizar a la población sobre prácticas sexuales seguras y medidas preventivas. Este enfoque integrado pretende mejorar la cobertura y la eficacia de las acciones de salud, promoviendo una atención más accesible y eficaz para las poblaciones clave.

## Data Availability

Los datos están disponibles en el OSF: https://www.doi.org/10.17605/OSF.IO/MJESK.
